# Modeling and optimization of CO_2_ mass transfer flux into Pz-KOH-CO_2_ system using RSM and ANN

**DOI:** 10.1038/s41598-023-30856-w

**Published:** 2023-03-10

**Authors:** Hassan Pashaei, Hossein Mashhadimoslem, Ahad Ghaemi

**Affiliations:** School of Chemical, Petroleum and Gas Engineering, Iran University of Science and Technology, Narmak, Tehran, 16846 Iran

**Keywords:** Engineering, Chemical engineering

## Abstract

In this research, artificial neural networks (ANN) and response surface methodology (RSM) were applied for modeling and optimization of carbon dioxide (CO_2_) absorption using KOH-Pz-CO_2_ system. In the RSM approach, the central composite design (CCD) describes the performance condition in accordance with the model using the least-squares technique. The experimental data was placed in second-order equations applying multivariate regressions and appraised applying analysis of variance (ANOVA). The *p*-value for all dependent variables was obtained to be less than 0.0001, indicating that all models were significant. Furthermore, the experimental values obtained for the mass transfer flux satisfactorily matched the model values. The *R*^*2*^ and Adj-*R*^*2*^ models are 0.9822 and 0.9795, respectively, which, it means that 98.22% of the variations for the *N*_CO2_ is explained by the independent variables. Since the RSM does not create any details about the quality of the solution acquired, the ANN method was applied as the global substitute model in optimization problems. The ANNs are versatile utensils that can be utilized to model and anticipate different non-linear and involved processes. This article addresses the validation and improvement of an ANN model and describes the most frequently applied experimental plans, about their restrictions and generic usages. Under different process conditions, the developed ANN weight matrix could successfully forecast the behavior of the CO_2_ absorption process. In addition, this study provides methods to specify the accuracy and importance of model fitting for both methodologies explained herein. The MSE values for the best integrated MLP and RBF models for the mass transfer flux were 0.00019 and 0.00048 in 100 epochs, respectively.

## Introduction

Universal warming and environmental destruction associated with the release of CO_2_, the most important greenhouse gas (GHG), have long been known as a significant threat to the future of the environment^1–6^. Several CO_2_ capture techniques, such as chemical and physical solvent absorption, membrane purification, cryogenic distillation, catalytic conversion, and adsorption processes, have been proposed for the separation and regeneration of CO_2_ emitted in the atmosphere^7–9^. The most widely applied technique for CO_2_ removal is the absorption of various solvent types^10,11^. Choosing an economically solvent system includes two essential criteria: high CO_2_ selectivity over other gases such as CH_4_ and N_2_, and high CO_2_ solubility. Furthermore, there is minimal solvent loss, low volatility, and a low energy requirement for solvent regeneration^12^, high thermal stability, high reaction rate, long-term stability, and high thermal stability are significant in the CO_2_ capture process^13^. The piperazine (Pz) solvent has many useful properties, including high CO_2_ capture capacity, fast CO_2_ absorption rate^14–16^, low regeneration energy and thermal damage, low oxidative damage, and low corrosion compared to MEA, which is widely used, DEA, and another alkyl amine^11,17,18^. To increase the fundamental ability and improve some of the Pz properties, modified absorbents are provided by adding various amines and metal oxides to the solution^19^. Modified absorbents have a high capacity for absorption, CO_2_ selectivity, and affordability in comparison to base fluid, and regeneration energy^20^. Saturation with potassium hydroxide (KOH) is more impressive in modifying the Pz solution, because KOH is low-cost, less corrosive rate, and is environmentally friendly. Many studies have been performed using Piperazine in an attempt to find effective absorbents for CO_2_ capture. Moiolo et al. used MDEA and Pz solutions for CO_2_ absorption and used ASPEN PLUS software for thermodynamic decomposition with the Electrolyte-NRTL technique for systems combining Pz-CO_2_-MDEA aqueous solutions^21^. They have analyzed the interaction effect between ion pairs and Pz or MDEA molecular species. Xu et al. proposed a thermodynamic model and studied the Pz concentration effect on CO_2_ absorption in MDEA solution^22^. The results demonstrated that Pz is useful for CO_2_ loading in the Pz-MDEA-H_2_O system. Liu et al. investigated the CO_2_ solubility in the Pz-MDEA-H_2_O system in a wide range of Pz concentrations (0.36–1.36 M) and temperatures (303‒363 K)^23^. Bishnoi and Rochelle studied the vapor–liquid equilibrium and CO_2_ solubility in the Pz-MDEA-H_2_O system using a wetted-wall column^24^. Bottger et al. published results on the solubility of CO_2_ in three different solutions of the Pz-MDEA-H_2_O system^25^. Furthermore, Speyer et al. investigated the CO_2_ solubility at low gas loadings of 313–393 K^26^. Najibi, and Maleki presented new experimental VLE data at different temperatures and pressures ranging from 363–423 K to 26.3–204.3 kPa, respectively^27^. Halim et al. investigated the performance of CO_2_ absorption at low pressure in Pz-MEA-H_2_O and Pz-AMP-H_2_O systems^28^. Ume et al. investigated the reaction kinetics of CO_2_ in the combination Pz with AEPD^29^. They noted that the absorption rate was significantly higher in the Pz-AEPD-H_2_O system in comparison to the base fluid.

To optimize the CO_2_ photo-reduction process, one factor at a time (OFAT) and trial-and-error optimization methods can be used^30^. The main disadvantage of the OFAT method is that it cannot estimate interactions and cannot explain the full effects of the parameters on the process^31,32^. Another disadvantage is that it cannot perform optimal agent setting, requires more runs for the same accuracy, which means more time, and incurs additional costs due to reagent costs^33,34^. In general, the primary goal of optimization is to develop and improve system performance, as well as to increase process efficiency while decreasing costs^35^. Recently, artificial neural networks (ANNs) and response surface methodology (RSM) techniques have been commonly used to simulate and optimize the CO_2_ absorption in an amine-based fluid.

The neural networks act like “black boxes” and can be easily applied to pattern recognition^36^ and predict different parameters as a function of different variables^37,38^. Neural network-based models are experimental in nature. The most significant benefits of ANNs modeling are: (i) the ease of computational efficiency; (ii) the ability to learn from examples^39^; (iii) the fact that prior knowledge of the relationships between variables is not required^40,41^; (iv) the ability to use it in complex non-linear processes where non-linearity can be more consistent with the data^42^; and (v) the ease of manipulation^43^. The original purpose of ANNs-based calculation (neuro-calculation) is to extend mathematical algorithms that can learn ANNs by imitating data processing and knowledge in the human brain^44^.

Response surface methodology (RSM), at first explained by Box and Wilson^45^, has since been widely applied as a method for designing experiments, for the approximation of the effects of numerous independent factors with their interactions on the observed response, and as an optimizing condition^46^. Latterly, RSM has been used to reduce the essential experimental data to achieve the best operating conditions for optimal response in multiple chemical processes^47–49^.

RSM is a set of statistical^50^ and mathematical techniques for optimizing numerous independent variables with fewer experimental tests^51–53^. RSM can help to study the interaction of process variables and create a mathematical model that precisely explains the general process. The most visible and impressive design used in the RSM to adapt a model using the least-squares technique is the central composite design (CCD)^54,55^. Compared to the Doehelt (DD) and Box–Behnken designs (BBD), the CCD offers advantages such as the need for only a few test points for its high application and performance^56^. Recently, RSM has been widely applied to appraise the results and performance of operations, even in many industrial processes^57^.

Several studies have been presented for the use of ANN and RSM for modeling and simulating absorption processes^49,58–60^. Nuchitprasittichai and Cremaschi used RSM to simulate the CO_2_ removal operation in an amine-based solution in order to identify the design variables and optimal process limitations while keeping the process cost low^61^. They also compared the RSM result in modality to an ANN prediction and concluded that the RSM technique could estimate the optimal solution information, which is similar to the solution obtained with ANN^36^. Also, in another study, a prediction algorithm for evaluating the suitable sample size by ANNs is presented as a substitute model^62^. RSM was used by Morero et al. to evaluate the performance of various solvents in the absorption/stripping process^63^. The effects of design variables such as pressure, temperature, flow rate, and CO_2_ concentration and their effects on energy cost and CO_2_ removal were investigated. Babamohammadi et al. used the CCD method to provide a CO_2_ solubility model in a mixed solution based on the concentration of MEA and glycerol, gas flow rate, and temperature^64^.

Sipocz et al. used a multilayer feed-forward form of ANN for CO_2_ capture^65^. They validated and developed the model into a conventional heat and mass balance program. In addition, they showed that the ANN model could produce the results of a precision process simulator in a fraction of the time. Basheer and Hajmeer^66^ applied ANN-based neurocomputing to the ANNs model as a practical guide and toolkit. ANNs were compared with statistical regression, and expert systems and their limitations and advantages were stated. Wu et al. investigated the nature of relationships between the main parameters using ANN approaches and statistical analysis for CO_2_ absorption from an amine-based solution^67^. In another study, these researchers analyzed the data for the amine-based CO_2_ capture domain^68^. They used the analysis process to clarify the relative importance of the parameters, including the use of ANN to model the relationship between the parameters and sensitivity analysis.

Zhou et al. combined two different approaches to ANN with sensitivity analysis (SA)^39^. In the ANN and SA combination method, four neural network models were developed, and the sensitivity analysis method has been used to express the relative order of importance of the parameters. The results showed that the concentration of amine is the most influential parameter in achieving the target performance^69,70^. Table 1 shows last literatures on using RSM models to the performance of CO_2_ capture.

**Table 1:** Some studies on the performance of *CO*_*2*_ capture using *RSM* techniques.

Sorbent	Variables	Response	Method	Model	R^2^, Adj-R^2^	References
DEA, TEPA, MSU-F	C_DEA_, C_TEPA_,C_MECH_	$q_{{CO_{2} }}$	Full factorial, CCD	$$\begin{gathered} Y = \, 5.62192 \, + \, 0.0830368X_{1} {-} \, 0.0345798X_{2} {-} \, 0.0208987X_{3} {-} \, 0.295X_{1} X_{2} {-} \, \hfill \\ 0.215X_{1} X_{3} {-} \, 0.1375X_{2} X_{3} {-} \, 0.156328X_{1}^{2} {-} \, 0.619369X_{2}^{2} {-} \, 0.140422X_{3}^{2} \hfill \\ \end{gathered}$$	0.953	^58^
Amine	T, P, Q_g_, and $P_{{\text{CO}_{2} }}$	T_rise_	CCD	$$\begin{gathered} T_{rise} = - 29.789 + 0.081T + 0.93P + 1.95P_{{CO_{2} }} + 0.37Q_{g} - 0.00493P^{2} \hfill \\ - 0.015P_{{CO_{2} }}^{2} + 0.00503TP + 0.1TP_{{CO_{2} }} - 0.00813TQ_{g} {-}0.0604PP_{{CO_{2} }} - 0.00249PQ_{g} \hfill \\ \end{gathered}$$	0.989, n.d	^71^
NaCl	T, Q_g_, C_B_, and S	%R	*CCD*	$$\begin{gathered} \% R = \, 60.55 - 0.328X_{1} + 3.118X_{2} + 0.0833X_{3} + 33.44X_{4} - 0.01844X_{1}^{2} \hfill \\ {-}0.1002X_{2}^{2} {-}0.0003X_{3}^{2} {-}20.55X_{4}^{2} + 0.03632X_{1} X_{2} + 0.307X_{1} X_{4} \hfill \\ \end{gathered}$$	0.988, n.d	^72^
NaCl	T, Q_g_, and M_r_	%R	*CCD*	$$\begin{gathered} \% R = \, 97.9 - 1.36T - 49.3Q_{g} - 10.5M_{r} - 0.0153T^{2} - 8.65Q_{g}^{2} \hfill \\ - 0.89M_{r}^{2} + 0.127TQ_{g} - 0.278TM_{r} + 21.47Q_{g} M_{r} \hfill \\ \end{gathered}$$	0.994, close to 1	^73^
Water	T, C_CO2_, Gas/liquid ratio,$P_{{\text{CO}_{2} }}$	%R	*CCD*	$$\begin{gathered} Q \times 10^{4} = \, - 8.71 - 30.71X_{1} - 0.63X_{2} + 11.16X_{3} + 1.47X_{4} \hfill \\ + 32.82X_{1}^{2} + 0.01X_{2}^{2} - 4.88X_{3}^{2} + 1.61X_{1} X_{2} + 24.34X_{1} X_{3} \hfill \\ - 1.65X_{1} X_{4} + 0.45X_{2} X_{3} - 0.02X_{2} X_{4} - 0.74X_{3} X_{4} \hfill \\ \end{gathered}$$	0.982, 0.972	^74^
Activated carbon	T,$P_{{\text{CO}_{2} }}$	$q_{{\text{CO}_{2} }}$, t_b_	Full factorial	$Q = 2.146 - 0.048T + 2.128P_{{CO_{2} }} - 0.106P_{{CO_{2} }}^{2} + 0.0003T^{2} - 0.0109T \times P_{{CO_{2} }}$	0.999, 0.998	^75^
				$t_{b} = 5.394 - 0.056T + 0.0003T^{2} - 0.017T \times P_{{CO_{2} }} + 0.981P_{{CO_{2} }} + 0.516P_{{CO_{2} }}^{2}$	0.990, 0.984	
MEA	T, Q_l_, Q_reb_, solvent loading	%R	CCD	$$\begin{gathered} \% R = \, 84.77 - 4.64A4.63B - 3.28C3.9D - 3.28E + 0.73AB - 0.5AC \hfill \\ + 0.59AD - 0.56AE + 0.81BC - 0.74BD + 0.53BE + 0.5CD - 0.54CE \hfill \\ + 0.5DE - 0.6B^{2} - 0.3C^{2} - 0.53D^{2} - 0.37E^{2} \hfill \\ \end{gathered}$$	0.974, 0.989	^76^
MEA	Q_l_, C_MEA_, and P	CO_2_ purity	n.d	$$\begin{gathered} Y \times 10^{4} = 9900 - 7.282A - 2.166B - 5.130C - 1.433D + 20.253E \hfill \\ + 0.0.04409AD + 3.540AE - 3.049BE + 2.044CE - 2.650DE \hfill \\ \end{gathered}$$	n.d., n.d	^77^
HMPD, AEEA	$$P_{{CO_{2} }}$$, C_HMPD_, C_AEEA_	$$\alpha_{{CO_{2} }}$$, absorption rate	n.d	$$\begin{gathered} (CO_{2} loading)^{1.78} = 9.5268 - 0.6534A - 0.15606B \hfill \\ - 0.042156C + 0.04238A^{2} + 5.04679 \times 10^{ - 5} C^{2} \hfill \\ - 2.88344 \times 10^{ - 7} D^{2} + 1.72214 \times 10^{3} D + 0.035597A \times B \hfill \\ + 7.89928 \times 10^{ - 4} A \times C - 2.30338 \times 10^{6} C \times D \hfill \\ \end{gathered}$$$$\begin{gathered} Ln(R_{A} ) = - 5.7302 + 0.6334A + 1.8444B + 5.1935 \times 10^{ - 3} C \hfill \\ + 5.547 \times 10^{ - 4} D - 0.1278A \times B + 5.2492 \times 10^{ - 5} A \times D \hfill \\ - 0.16196A^{2} - 0.53106B^{2} - 1.62695 \times 10^{ - 7} D^{2} \hfill \\ \end{gathered}$$	0.962, 0.949 0.969, 0.967	^78^
MEA, Glycerol	$C_{MEA} ,C_{glycerol}$ T, Q_g_	$\alpha_{{CO_{2} }}$	CCD	$$\begin{gathered} {\text{CO}}_{2} {\text{ Solubility = }} - 56.737 + 15.15341{\text{A + }}12.246{\text{B}} + 0.376{\text{C}} - 0.0665{\text{D}} \hfill \\ - 1.743A \times B + 7.691 \times 10^{ - 3} B \times D + 2.2110A^{2} + 1.196{\text{B}}^{2} \hfill \\ \end{gathered}$$	0.953, 0.935	^64^
DGA, DEPG	_P, T,_$P_{{CO_{2} }}$	$q_{{CO_{2} }}$, Q_reb_, Q_cooling_	CCD	$Power requirement = + 76783 + 49159A + 33010B + 25703AB$	0.999, 0.998	^63^
				$Cooling \, duty = + 80575.1 + 50957.3A + 32256.2B - 4236.4C + 25294.1AB$	0.997, 0.995	
				${\text{Re}} {\text{cov}} ered \, CH_{4} = + 97.4{-}3.9B + 2.3C - 0.4D + 2.1BC + 0.6CD$	0.977, 0.944	
				$Captured \, CO_{2} = + \, 89.6 + 13.9A + 27.1B - 9.1C - 23.3B^{2}$	0.961, 0.907	
MEA, DGA DEA, MDEA, TEA	_ns, absorber, ns,stripper, Camine,_ Q_reb_, T_stripper_	CO_2_ capture	Full factorial, Box–Behnken	_n.d_	n.d	^61^
Aminated activated carbons	_Tamination_	$q_{{CO_{2} }} ,q_{{CO_{2} ,des}}$	CCD	$$\begin{gathered} Y_{S} = 27.121 + 2.962x_{1} + 0.569x_{2} + 0.787x_{3} - 1.237x_{1}^{2} \hfill \\ - 1.486x_{2}^{2} - \, 0.292x_{1} x_{2} + \, 0.83x_{1} x_{3} \hfill \\ \end{gathered}$$	0.976, 0.964	^60^
				$$\begin{gathered} Y_{D} = 93.70 - 2.63x_{1} - 0.478x_{2} - 0.253x_{3} + 1.357x_{1}^{2} \hfill \\ + 0.859x_{2}^{2} + 0.192x_{1} x_{2} - 0.328x_{1} x_{3} \hfill \\ \end{gathered}$$	0.988, 0.982	
Activated carbon	P_des_,T_des_, P/F ratiodes	$q_{{CO_{2} }}$	CCD	$q_{{CO_{2} }} = 2.647 + 0.001T_{des} + 0.268P/F \, ratio - 0.139\left( {P/F \, ratio} \right)^{2}$	0.879, 0.746	^79^
				$CO_{2} \, recovery = 93.377 + 0.029T_{des} + 9.447P/F \, ratio - 4.891\left( {P/F \, ratio} \right)^{2}$	0.880, 0.746	
				$Productivity = 7.942 + 0.003T_{des} + 0.804P/F \, ratio - 0.416\left( {P/F \, ratio} \right)^{2}$	0.880, 0.746	
				$H_{2} \, purity = 97.2066 + 0.0123T_{des} + 4.0052P/F \, ratio - 2.0771\left( {P/F \, ratio} \right)^{2}$	0.878, 0.742	
				$CO_{2} \, purity = 90.7025 - 0.0949T_{des} + 1.4473P_{des} - 12.9794P/F \, ratio$	0.867, 0.720	
				$r_{max} \, _{des} = 29.100 + 0.208T_{des} - 3.436P_{des} - 11.930P/F \, ratio$	0.804, 0.586	

n.d. = not define.

In the present study, the composing effects of KOH and Pz concentrations, temperature, CO_2_ loading, gas film, liquid side mass transfer coefficient, partial pressure of CO_2_, and equilibrium CO_2_ partial pressure of the bulk solution on the CO_2_ mass transfer flux were investigated. Because the design and statistical models can be applied for modeling and optimization of the process^50,80–82^, the RSM and ANNs were used. In the RSM, the experimental data were examined by fitting a second-order polynomial model, which was statistically confirmed by performing an analysis of variance (ANOVA) and a lack-of-fit test to assess the importance of the model. Moreover, the objective was to demonstrate that the fitted RSM could serve as a tool to perform and optimize control factors in CO_2_ absorption (mass transfer flux). The goal of our work with the ANN approach was to explain the nature of relationships between the main process parameters using data modeling and analysis approaches. The ANN models were developed based on the relationships between parameters based on the operational data, and the sensitivity analysis method was used to reveal the order of importance among the input parameters for each model.

## Experimental setup

**Figure 1:**
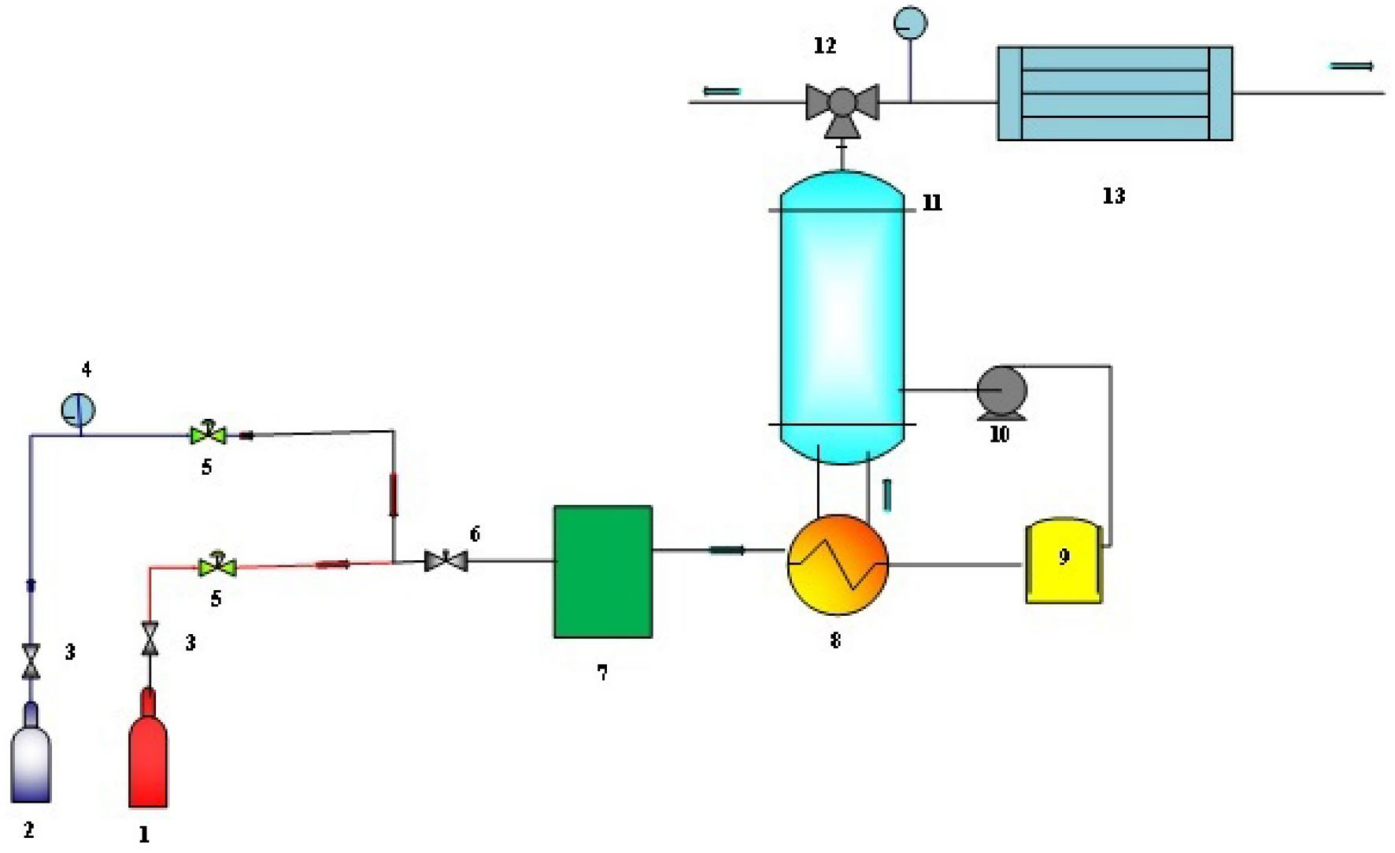
Experimental setup employed in the CO_2_ absorption experiments: (1) CO_2_ cylinder; (2) N_2_ cylinder; (3) Ball valve; (4) Pressure gauge; (5) Flow controller; (6) gate valve; (7) Saturation tank; (8) Heater; (9) Solution tank; (10) Pump; (11) wetted-wall column; (12) Pressure controller; (13) Condenser^75^.

The tests were performed in a wetted-wall column shown in Fig. 1. The experimental equipment contains a stainless-steel tube portion with 1.26 cm outer diameter and height of 9.1 cm, CO_2_ and N_2_ cylinders with a regulator, and a gas flow meter, pump, heater, and condenser, and two solution reservoirs and saturation tanks. The total contact area and longitudinal area were 38.52 cm^2^, and 36.03 cm^2^, respectively. To prepare the liquid and gas contact box, the column is surrounded by a thick-walled glass tube with an outside diameter of 2.54 cm^83^. The hydraulic diameter and cross-sectional area were 0.44 cm and 1.30 cm^2^, respectively. Cullinane's experimental data was used to evaluate the ANNs and RSM parameters^83^.

## Theory

### Response surface methodology

RSM is one of the most appropriate tools for analyzing, evaluating, and modeling the outcome of various operating interactive variables. RSM was first extended by Box and Behnken^84,85^. This statement was originated from the graphical representation created since its adaptation to the mathematical model and has been widely used in the field of chemometrics. The RSM model includes mathematical groups and statistical methods derived from experimental models to fit the experimental data about testing settings. For this purpose, second-degree polynomial functions were used to illustrate the process^86^.

**Figure 2:**
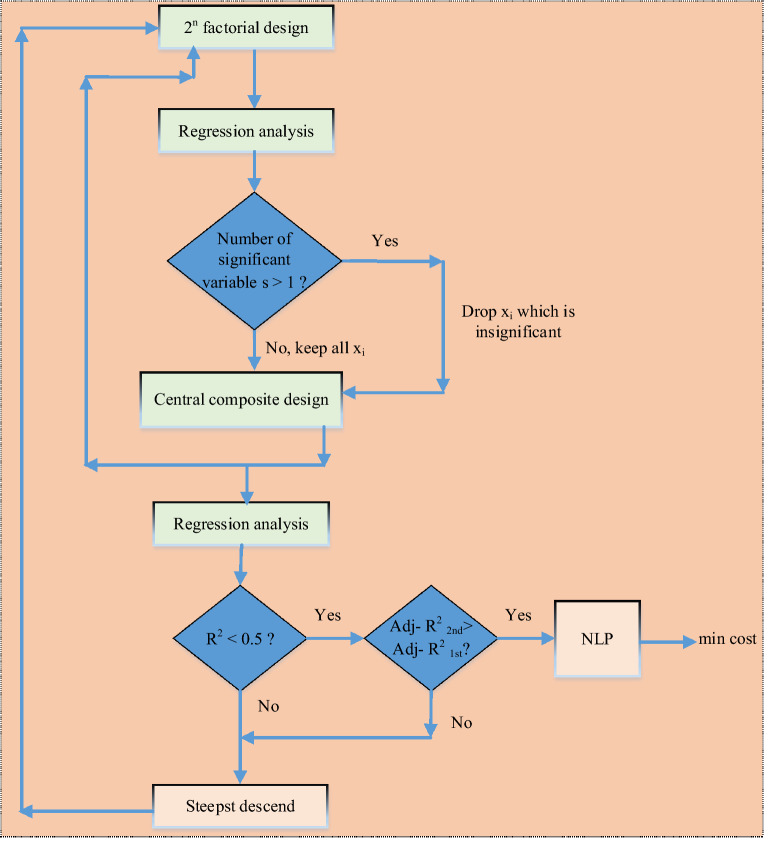
The scheme of study for RSM.

In this study, CO_2_ mass transfer flux was investigated using RSM. The decision variables are the Pz and KOH concentrations, the temperature of the solution, the CO_2_ loading, the liquid and gas phase mass transfer coefficients, the CO_2_ partial pressure of the gas bulk_,_ and the equilibrium CO_2_ partial pressure of the bulk solution. The aim is to maximize CO_2_ mass transfer flux. RSM uses the first- or second-order local regression models of the target function to conduct the optimization search by providing an appropriate direction for the performance of the functions^87^. Optimization can be apportioned into six steps as follows: (1) selection of possible factors and responses^88^; (2) choose of experimental design strategy^82^; (3) performance of experiments and obtaining results^89^; (4) model fit with the experimental data^90^; (5) obtain response diagrams and confirmation from the model (ANOVA); and (6) obtain the optimal conditions. Figure 2 depicts the optimization algorithm used in this case.

The algorithm consists of two major phases. In the first phase, the process simulation is performed using a complete experimental design of factorial determination variables (a 2^8^ design of factorial), and the mass transfer flux is calculated for each run. The first data is set according to the operating conditions presented in the other works^83^. The initial variables of each solvent are estimated from the usual range used in the literature^83^. The range of changes in design variables and responses is shown in Supplementary Table S1. In the present work, the central composite design (CCD) was used to optimize the useful variables and parameters and fit a quadratic surface, as well as to explore the interaction between parameters. Generally, the CCD consists 2^n^ factorial runs with 2^n^ axial and n_c_ center run (ten repeats). The relationship between the actual and coded values of the variables is shown in Supplementary Table S2. A 2^8^ full factorial CCD was used, with 256 factorial points, 16 axial points, and ten replicates at the center points. So, 282 experiments were obtained from Eq. (1).

1 $$N = 2^{n} + 2n + n_{c} = 2^{8} + 2 \times 8 + 10 = 282$$

Before regression analysis, the intention variables (x_i_) are encoded at intervals of (− 1, 1), called coded variables. If $\xi_{i}$ represents the natural variable, the relationship between the natural variables and coded variables is given by Eq. (2).

2 $$x_{i} = \frac{{\xi_{i} - \xi_{i}^{0} }}{{\Delta \xi_{i} }}\quad {\text{for}}\quad i = \, 1, \, 2, \, \ldots , \, n\;{\text{factors}}$$

where $\xi_{i}^{0}$ is the natural variable that located in the domain center and $\Delta \xi_{i}$ is the variation of the natural variable relevant with the change of one unit of the coded variable. It is calculated as:

3 $$\Delta \xi_{i} = \frac{{\xi_{i}^{\max } - \xi_{i}^{\min } }}{2}$$

The minimum and maximum values of the distinct variables are chosen to confirm that the corresponding coded variables applied for the 2^8^ factorial designs and CCD are integer values.

In the second phase, the process simulation is run according to the CCD of the numerous variables. The CCD is a second-order design class that avoids severe condition testing because it does not include the conditions where all factors are at their lowest or highest levels^56^. One of the disadvantages of using RSM with definite simulations is the lack of change in the results of the repetitive experiments at the center point; therefore, this system does not include a pure experimental error variance to simplify the switch between the second-order and first-order models. Instead, we used the *R*^2^ statistics and adjusted *R*^2^ (Adj-*R*^2^) statistics to evaluate the suitability of the first-order and second-order models to the data collected from the simulation. The step size in the steepest descend path used by the first-order model (Eq. (4)) is $\Delta x_{i} = \beta_{i} /\beta_{j} /\Delta x_{j}$ where $\beta_{j}$ is the coefficient of the largest absolute regression. The step size is reduced by half until it is reduced to the target performance.

4 $$Y = \beta_{0} + \sum\limits_{i = 1} {\beta_{i} } X_{i}$$

On the other hand, if it is found that the data according to *R*^2^ statistic cannot be accurately represented with the first-order model; the adj-*R*^2^ statistic is used to affirm that second-order factors (Eq. (5)) have considerable effects on response prediction^61,91^.

5 $$Y = \beta_{0} + \sum\limits_{i = 1} {\beta_{i} } X_{i} + \sum\limits_{i = 1} {\beta_{ii} } X_{i}^{2} + \sum\limits_{i = 1} {\sum\limits_{j = i + 1} {\beta_{ij} } X_{i} } X_{j} + \varepsilon \quad {\text{x}}_{{\text{i}}} \;{\text{for}}\quad {\text{i}} = {1},{2}, \, . \, . \, .,{\text{ n}}\;\;{\text{factors}}$$

The nonlinear programming (NLP) model related to this issue is presented as follow:

6 $$Y = \beta_{0} + \sum\limits_{i = 1} {\beta_{i} } X_{i} + \sum\limits_{i = 1} {\beta_{ii} } X_{i}^{2} + \sum\limits_{i = 1} {\sum\limits_{j = i + 1} {\beta_{ij} } X_{i} } X_{j} \quad - {1} \le {\text{xi}} \le {1}\;{\text{for}}\;{\text{i}} = {1},{2}, \, . \, . \, .,{\text{ n}}\;{\text{factors}}$$

where, *Y* is a function of the predicted response (i.e., CO_2_ mass transfer flux),* X*_*i*_ and *X*_*j*_ indicate the model value of variables *i*, and *j*, *β*_*0*_ is the offset, *β*_*i*_ and *β*_*ii*_ (the effect of interaction coefficient) represent the coefficients of the linear and quadratic parameters, respectively, and ε represents the residual associated with the experiments^49,89^. Multiple regression analysis of the experimental data according to Eq. (5) was performed by the least-squares method, which produces *β* coefficients with the least possible residuals. The CO_2_ mass transfer flux was optimized using RSM. The optimal decision variables (i.e., Pz and KOH concentrations, temperature, loading, mass transfer coefficients, CO_2_ partial pressure) were created with the response optimizer. After getting data about each practical level of the selected scheme, it is necessary to study the behavior of points of similar response to fit a mathematical equation. This means their parameters *b* must be approximated from Eq. (5). Thus, in marking the matrix, Eq. (5) can be represented as^92^:

7 $$y_{m} x_{i} = X_{m} x_{n} b_{n} x_{1} + e_{m} x_{1}$$

where b and y are the model and response parameter vectors, respectively, *m* and *n* are the numbers of rows and columns of the matrices, respectively, *X* is the selective experimental design matrix, and* e* is the residual. Equation (7) is solved using a statistical method called the method of least squares (MLS) ^32^. A vector b can be computed as Eq. (8) in the following^36^:

8 $$b_{n - 1} = (X_{n - m}^{T} y_{m - i} )(X_{n - m}^{T} X_{m - n}^{{}} )^{ - 1}$$

The significance and fitness of the model were also confirmed using Analysis of Variance (ANOVA) in the Design-Expert software. The central premise of ANOVA is that each measured value is a function of three components consists overall mean value (α), measured factors’ effects on the system response (β), and residual error (ε).

9 $$Y_{i} = \alpha_{i} + \beta_{i} + \varepsilon_{i}$$

By converting this model, the following equation can be obtained for the residual error.

10 $$\varepsilon_{i} = Y_{i} - \alpha_{i} - \beta_{i}$$

Further variation prepares an equation for the sum of squares for the residual errors.

11 $${\text{RSS}} = \sum ( Y_{i} - \alpha_{i} - \beta_{i} )^{2}$$

In ANOVA, data set changes are evaluated by surveying their dispersion. Evaluate the deviation (*d*_*i*_) in which each observation (*y*_*i*_) or its iteration (*y*_*ij*_) is present concerning the medium (ȳ), or, more precisely, the square of this deviation is represented in Eq. (12):

12 $$d_{i}^{2} = (y_{ij} - \overline{y} )^{2}$$

The sum of the square for all examination deviations about the medium is called the total sum of the square (*SS*_tot_); it can be fragmented into the sum of the square according to the regression (*SS*_reg_) and the sum of the square according to the residuals generated by the model (*SS*_res_), as shown below ^89^:

13 $$SS_{{{\text{tot}}}} = SS_{{{\text{res}}}} + SS_{{{\text{reg}}}}$$

As the center point is replicated, the pure error associated with the repetition can be estimated. Therefore, the sum of the square for residuals can be divided into two sums of the square for pure error (*SS*_pe_) and lack of fit (*SS*_lof_) parts, as represented below^89^:

14 $$SS_{{{\text{res}}}} = SS_{{{\text{pe}}}} + SS_{{{\text{lof}}}}$$

By dividing the sum square for each square's source (pure, residual, regression, total, and lack of fit error) by their degree of freedom (*d.f.*), the “media of the square” (MS) is obtained. The numbers representing the degree of freedom for these sources are represented in supplementary Table S3^85,86^.

To determine the importance of parameters, the *p*-value test with a minimum of 95% confidence was used for the experimental results. The goodness of fit of the polynomial model was represented by the coefficients of specification, the absolute average deviation (AAD), *R*^*2*^, and *adj*-*R*^*2*^ through Eqs. (15)–(17). AAD should be as low as possible between the predicted and observed values, and* adj-R*^*2*^ should be close to 1.0. AAD shows the deviations between the experimental and calculated quantities, and *adj-R*^*2*^ displays the ratio of data variables specified by the model^32^.

15 $${\text{AAD}}\% = 100\left[ {\sum\limits_{i = 1}^{n} {(\left| {y_{i,\exp } - y_{i,cal} } \right|/y_{i,\exp } )} } \right]/n$$

where n is the experiment numbers and y_i,cal_ and y_i,exp_, are the calculated and experimental responses, respectively.

16 $$R^{2} = 1 - \frac{{SS_{res} }}{{SS_{\bmod el} + SS_{res} }}$$

17 $$Adj - R^{2} = 1 - \frac{{SS_{res} /d.f._{res} }}{{(SS_{\bmod el} + SS_{res} )/(d.f._{\bmod el} + d.f._{res} )}}$$

Here, *SS* is the sum of squares and, *DF* is the degrees of freedom. Moreover, some parameters are used to influence diagnostics through the following equations presented in Table 2.

**Table 2:** Some parameters definitions and their application in the *RSM-CCD* model.

Definition	Equation	Objective of study
Small subset	$\hat{\overline{Y}} = X\hat{\beta }$**,**$e = Y - \hat{\overline{Y}}$	- Has a asymmetrical result on the regression
		- The predicting variable depends more on the impression subset than the majority information
Leverage	$H = X(X^{T} X)^{ - 1} X^{T} = \left[ {h_{ij} } \right]$	- Shows the fraction of variance in the error along with the approximate point
		-The capability of a design point to represent model fit coefficients
		- A point that varies from 0–1, and indicates the design point effect on the model’s values
		- H = 1 means that the experiments and predicted value are quite equal, i.e., the residual will be zero
Internally Studentized Residual	$r_{i} = \frac{{e_{i} }}{{\hat{\sigma }\sqrt {1 - h_{ii} } }}$	- Distinguishing between predicted and actual values
Externally Studentized Residual	$\hat{\sigma }^{2}_{( - i)} = \frac{{(n - p).\sigma^{2} - \frac{{e_{i}^{2} }}{{(1 - h_{ii} )}}}}{{\sigma \sqrt {1 - h_{ii} } }}$	- By leaving each run, out of the surveying, predicted the response from the remaining runs
t-value	$t_{i} = \frac{{e_{i} }}{{\sqrt {\hat{\sigma }_{( - i)}^{2} (1 - h_{ii} )} }}$	- The number of standard deviations between the predicted and actual response values
		- Counts the number of standard deviations that separate the parameter estimate from zero
DFFITS	$DFFITS = \frac{{\hat{\overline{Y}} - \hat{Y}_{( - i)} }}{{\sqrt {\hat{\sigma }_{( - i)}^{2} } .h}}$	- The studentized difference between the predicted and observed value i
	$$\hat{Y}_{( - i)} = Y + \frac{e}{1 - h}$$	
DFBETAS	$DFBETAS_{j,( - i)} = \frac{{\hat{\beta }_{j} - \hat{\beta }_{j,( - i)} }}{{\sqrt {\hat{\sigma }_{( - i),i}^{2} .(X^{T} X)_{jj}^{ - 1} } }}$	-Shows the influence the i^th^ observation has on every regression coefficient
		- Is a square yield of a uniform leverage function and the i^th^ internally studentized residual
Cook’s distance	$D_{i} = \frac{{r_{i}^{2} }}{p + 1}(\frac{h}{1 - h})$	- Shows how well the model fits the *i*^th^ seeing *y*_*i*_ and an ingredient that measures the distance of that point from the rest of the data

Where,* e*, *y*_*i*_, and $\hat{\overline{Y}}$, and *e* are error value, the measured response data, and the predicted value from the model, respectively. X and H are the model matrix of n rows and p columns. n is the number of runs minus the one being left out and p is the number of terms in the model including the intercept.

### Artificial neural network

The neural network method (ANN) conforms to the human nerves and brain structure, and it has become trendy over the last two decades^93^. ANN is a computational and mathematical model that can simulate phenomena by taking patterns from biological neural networks. In the 1940s, the ANN structure was used to classify data for different subjects^94^. Researchers such as Grossberg, Widrow, Hopfield, and Rumelhart improved the ANN method in the 1980s^95–97^. The benefits of neural networks include their fast processing speed, relationship between input and output data, network compatibility, noise data response, fault tolerance, and learning^98^. Neurons are the smallest units of data processing. Numbers enter the neuron unit as the input and output data. The input signals of a cell are aggregated together. The aim of ANN is to acquire the suitable values of weights ($w$) for a given function ($f$). Each input ($x_{i}$) is multiplied by the corresponding weight factor ($w$), then sum of all the values is computed, and the bios value ($b$) or threshold is added to the sum of values. A summary of this process for the input data is expressed in Eq. (18).

18 $$net = \left( {\sum\limits_{i = 1}^{n} {\left( {\omega_{i} x_{i} } \right) + b} } \right)$$

The final yields are fed into a transfer function ($f$), then the Eq. (19) generates as values *(*$y$):

19 $$y = f(net)$$

Transfer functions are typically sloping, linear, step, or sigmoid ($S$ shape). The neural layers are made up of nerve cells, and the network is made up of one or more layers of neurons.

#### Multi-layer perceptron (MLP)

Learning ANN means finding algorithms for specifying the weighted relationships between neuron. One of the most popular neural networks used to create nonlinear mappings is the multi-layered perceptron. The MLP function method is based on Eq. (20). In this relation, $g$ is the output vector, and $\theta$, $w$, and $x^{k}$ indicates the threshold limit, the weighted vector of coefficients, and the input vector, respectively^99^. A multi-layer preceptor receives, processes, and indicates information via an input layer, one or more latent layers, and an output layer.

20 $$g = f(wx_{i}^{k} + \theta )$$

Supplementary Figure S1 shows the MLP neural network's structure, which includes the input layer, hidden multilayers, and output layer. In this approach, the training algorithm process is divided into two parts: the forward pass and the backward pass, to lower the average squares of general error. The input vector is fed into the network in the forward pass, and the network output is generated, while the error is determined in the backward pass as the ratio between the network and experimental data outputs. This output moves from the existing layer to the back layer, then the weights in the input layer change. The weights are corrected so that the mean square of the total errors is minimized. Finally, the training process ends with weight correction and bias. In this study, the Bayesian training method is applied to solve the network, which can be used to train the NN feed-forward propagation algorithm. The amount of weights and biases is first assumed to be related to a distribution function with an unknown variance in this method^98,100^. The MLP neural network output can be developed as follows:

21 $$\gamma_{jk} = F_{k} \left( {\sum\limits_{i = 1}^{{N_{k - 1} }} {w_{ijk} \gamma_{i(k - 1)} + \beta_{jk} } } \right)$$

where $\gamma_{jk}$, and $\beta_{jk}$ are the neuron *j*’s output from *k*’s layer and bias weight for neuron *j* in layer *k*, respectively. The *w*_*ijk*_ is the link weights that were chosen at random at the start of the network training process, and *F*_*k*_ is the nonlinear activation transfer functions, which may take many various forms such as identity, bipolar sigmoid, binary step function, binary sigmoid, linear, and Gaussian functions^101^.

#### Radial basis function (RBF) network

For the first time, Brodhead and Lowe in 1998^102^ introduced the radial-based functions (RBF) network, which is a kind of forward-feed network with a single hidden layer. Although the RBF approach is similar to MLP, the definition of single hidden layer neurons differs significantly. The data from the input layers is gathered in the single hidden layer and transmitted to the Gaussian transfer function, causing the data to become nonlinear. The transfer functions between the input layer and the single hidden layer are nonlinear in the RBF neural network, whereas the transfer functions between the single hidden layer and the output layer are linear. The distance (geometric or Euclidean size) between both the input vector and the weights is measured by the hidden neurons in RBF networks. Linear combiners are the RBF output layer, as defined by Eq. (22).

22 $$f(x) = \sum\limits_{i = 1}^{N} {w_{ij} G(\left\| {x - c_{i} } \right\|} *b)$$

*N*, $w_{ij}$, *x*,$c_{i}$, and *b* are the number of training data sets, the weight associated with each hidden neuron, the input variable, the center points, and the bias parameters, respectively. Equation (23) yields the centralized response from the concealed location using a Gaussian function:

23 $$G(\left\| {x - c_{i} } \right\|*b) = \exp \left( {( - \frac{1}{{2\sigma_{i}^{2} }}(\left\| {x - c_{i} } \right\|*b)^{2} } \right)$$

where, $\sigma i$ is the Gaussian function's spread. Supplementary Figure S2 shows the RBF NN structure, which has an input layer that includes temperature, pressure, and concentration, a single hidden layer with neurons, and an output layer that includes.

#### Design procedure of ANN structure

The flowchart diagram for the ANN model design process is demonstrated in supplementary Figure S3. In the first phase, we have data collection. The thermodynamic and process parameters, such as the solution concentration of KOH and Pz, temperature, CO_2_ loadings, gas side mass transfer coefficient (*k*_*g*_), liquid film mass transfer coefficient (*k*_*l*_), the partial pressure of CO_2_ at the gas–liquid interface ($p_{{{\text{CO}}_{2} ,b}}$), and the equilibrium partial pressure of CO_2_ in bulk ($p_{{{\text{CO}}_{2} }}^{*}$) as input data and absorption flux of a gas into a liquid ($N_{{{\text{CO}}_{2} }}$) was used as targets data. In the next phase, the ANN model is defined by the input and output or target variables and parameters. The network architecture was developed in the following stage with the selection of learning algorithms using the input and target normalized data as machine language. In the fifth stage, the input and output data are rectified if required, and the ANN model training method is utilized, together with network training validation, to match the data and choose the training, including network input and output adjustment. The selected model is taught using a set of training data to maximize the model's performance by setting network parameters such as weight, bias, and threshold. The network validation data is enhanced using a set of data collected during the training, and the network testing process is guided by the test data. When generalizations have improved, the training procedure comes to an end. To compare the model outputs with the data set, statistical metrics such as the *R*^*2*^ value and the mean square error (MSE) are used to evaluate the trained model's performance. The optimum network pattern is chosen and developed in the final phase. Numerous setups were evaluated in the development of the MLP model, and the performance of the network was optimized by changing the number of hidden layers, the number of neurons in the hidden layers, and the network training algorithm to obtain the best network for predicting the absorption flux of a gas into a liquid ($N_{{{\text{CO}}_{2} }}$). How to select neurons in the RBF network is typically a trial-and-error process in which the learning algorithm begins with a large number of neurons in the single hidden layer and then reduces this number as much as possible. This reduction of neurons is associated with minimizing the proportional error, and when the optimized error is obtained from testing data, the training process of the algorithm ends.

## Result and discussion

### RSM-CCD approach

The RSM-CCD model was used to analyze various parameters affecting the performance of CO_2_ mass transfer flux. The independent variables are the different values that should be specified by the experiments. All of these independent variables were examined at five levels. Before using the RSM, an experimental design was chosen that determined what experiments should be performed in the experimental area under survey as a set of different combinations of the independent variable levels. In this work, the effects of eight variables, including KOH concentration (*X*_*1*_), Pz concentration (*X*_*2*_), temperature (*X*_*3*_), CO_2_ loading (*X*_*4*_), gas film mass transfer coefficient(*X*_*5*_), liquid side mass transfer coefficient (*X*_*6*_), the gas bulk partial pressure of CO_2_ (*X*_*7*_), and equilibrium CO_2_ partial pressure (*X*_*8*_) on CO_2_ mass transfer flux were investigated using RSM.

#### Analysis of variance (ANOVA)

The central composite responses are studied, and the analysis of variance (ANOVA) results are presented in Table 3. To find an appropriate model and evaluate the accuracy of the RSM method, various indicators such as the F-value and *p*-value are used. This comparison is performed using the value of Fisher's F-test as indicated in Table 3. According to ANOVA tables, *p*-values for the models of the CO_2_ mass transfer flux are less than 0.0001; therefore, the models are significant (*p*-value < 0.05). Besides, RSM analysis is used to generate semi-empirical correlations, which link process and response parameters. The effects of independent variables on $N_{{{\text{CO}}_{2} }}$ are presented in Eqs. (24) and (25) as the coefficients of the second-order polynomials, in terms of coded and actual factors.

24 $$\begin{aligned} {\text{Actual Equation}} \hfill \\ N_{{CO_{2} }} =& - 56.638 + 9.585X_{1} - 22.689X_{2} + 4.805X_{3} + 284.685X_{4} + 18.775X_{5} - 506.306X_{6} \hfill \\ &+ 0.027X_{7} - 0.024X_{8} + 1.427X_{1} X_{2} - 0.277X_{1} X_{3} - 34.675X_{1} X_{4} - 2.760X_{1} X_{5} + 47.172X_{1} X_{6} \hfill \\ &+ 4.10 \times 10^{ - 4} X_{1} X_{7} - 3.89 \times 10^{ - 4} X_{1} X_{8} - 0.995X_{2} X_{3} - 24.34X_{2} X_{4} - 0.385X_{2} X_{5} \hfill \\ &+ 116.039X_{2} X_{6} + 5.66 \times 10^{ + 4} X_{2} X_{7} - 14.10 \times 10^{ + 4} X_{2} X_{8} + 14.073X_{3} X_{4} + 0.289X_{3} X_{5} \hfill \\ &- 13.628X_{3} X_{6} + 1.90 \times 10^{ - 5} X_{3} X_{7} + 4.40 \times 10^{ - 5} X_{3} X_{8} - 6.237X_{4} X_{5} - 609.01X_{4} X_{6} \hfill \\ &- 0.0417X_{4} X_{7} + 0.037X_{4} X_{8} - 23.018X_{5} X_{6} - 3.58 \times 10^{ - 4} X_{5} X_{7} + 1.09 \times 10^{ - 4} X_{5} X_{8} \hfill \\ &+ 0.006X_{6} X_{7} - 0.012X_{6} X_{8} - 1.239 \times 10^{ - 7} X_{7} X_{8} - 1.114X_{1}^{2} + 0.159X_{2}^{2} + 0.052X_{3}^{2} \hfill \\ &+ 213.88X_{4}^{2} - 0.404X_{5}^{2} + 820.059X_{6}^{2} + 7.879 \times 10^{ - 9} X_{7}^{2} + 2.048 \times 10^{ - 7} X_{8}^{2} \hfill \\ \end{aligned}$$

25 $$\begin{aligned} {\text{Coded Equation}} \hfill \\ N_{{CO_{2} }} = &- 117.67 - 50.14X_{1} + 19.12X_{2} + 129.54X_{3} - 186.91X_{4} - 408.08X_{5} + 15.12X_{6} + 235.39X_{7} \hfill \\ &- 240.58X_{8} + 3.96X_{1} X_{2} - 26.03X_{1} X_{3} - 16X_{1} X_{4} - 80.86X_{1} X_{5} + 37.53X_{1} X_{6} + 22.8X_{1} X_{7} \hfill \\ &- 13.22X_{1} X_{8} - 75.82X_{2} X_{3} - 9.11X_{2} X_{4} - 9.14X_{2} X_{5} + 74.85X_{2} X_{6} + 25.5X_{2} X_{7} - 38.81X_{2} X_{8} \hfill \\ &+ 51.63X_{3} X_{4} + 232.19X_{3} X_{5} - 1297.69X_{3} X_{6} + 29.51X_{3} X_{7} + 41.21X_{3} X_{8} - 24.64X_{4} X_{5} \hfill \\ &- 65.34X_{4} X_{6} - 312.15X_{4} X_{7} + 168.76X_{4} X_{8} - 156.73X_{5} X_{6} - 170.1X_{5} X_{7} + 31.59X_{5} X_{8} + 82.9X_{6} X_{7} \hfill \\ &- 91.71X_{6} X_{8} - 73.29X_{7} X_{8} - 3.81X_{1}^{2} + 0.357X_{2}^{2} + 133.19X_{3}^{2} + 13.31X_{4}^{2} - 101.39X_{5}^{2} + 151.63X_{6}^{2} \hfill \\ &+ 7.1X_{7}^{2} + 69.02X_{8}^{2} \hfill \\ \end{aligned}$$

**Table 3:** ANOVA for response surface quadratic model.

Source	Sum of squares	Degree of freedom	Mean square	F-value	*p*-value
Model	1.216 × 10^6^	44	27,637.72	360.43	< 0.0001
Residual	22,006.89.15	287	76.68	6.38	< 0.0001
R^2^			0.9822		
Adj.R^2^			0.9795		
Pred.R^2^			0.9740		
Adeq. Precision			135.873		
Lack of Fit	5.69	294	0.7116	0.195	0.9466
Pure Error	3.64	0	3.64		
Cor Total	1.238 × 10^6^	332	4791.69		

**Figure 3:**
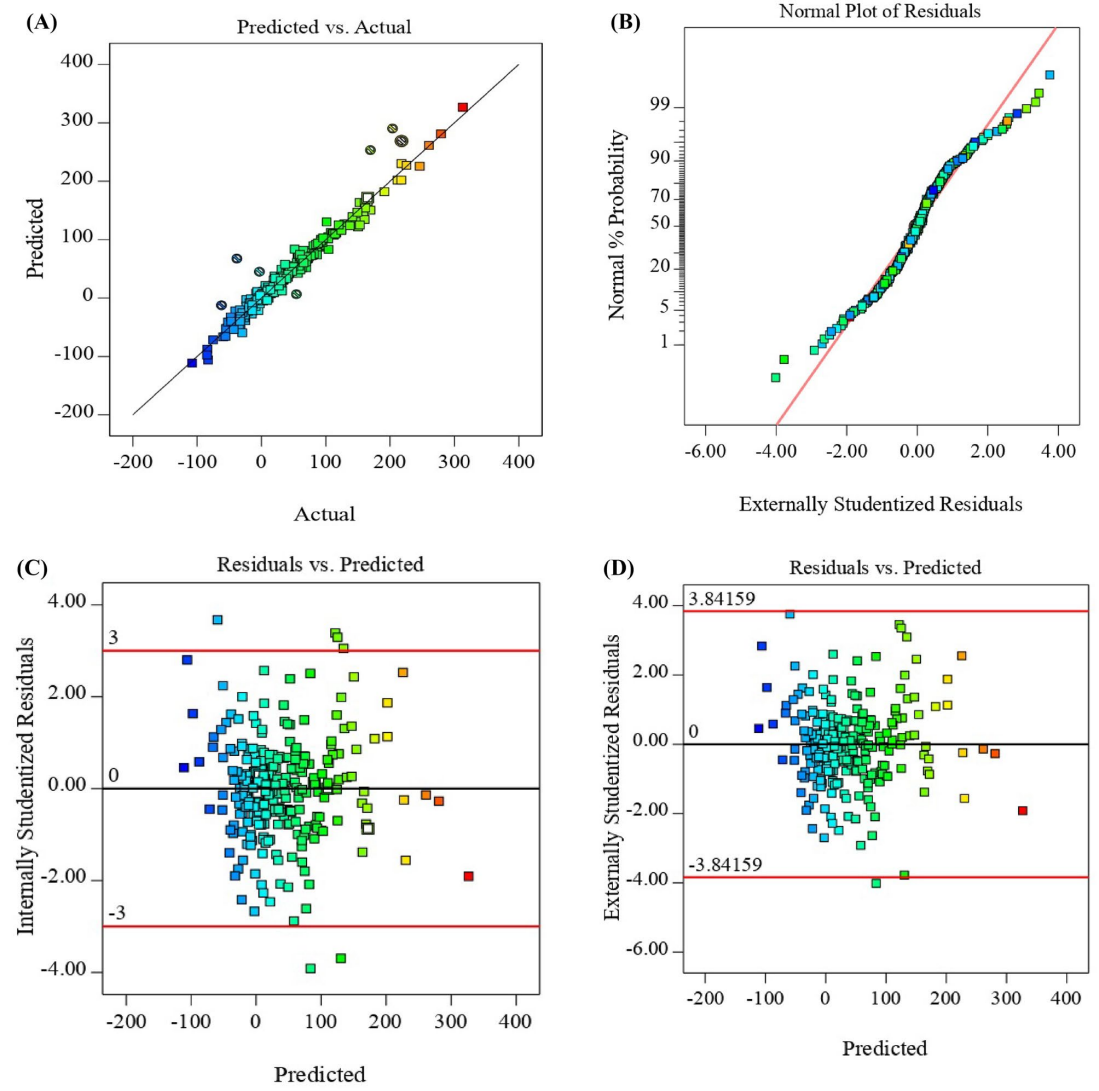
The CCD Predicted value of mass transfer flux; (**A**) actual absorption vs. predicted, (**B**) externally studentized residuals vs. normal plot (**C**) predicted vs. internally studentized residuals, and (**D**) predicted vs. externally studentized residuals.

The Eqs. (24) and (25) can be used to predict the response for given levels of each factor. The coded equation is useful for identifying the relative effect of the factors by comparing the factor coefficients. The actual equation should not be used to determine the relative effect of each factor. Because the coefficients are scaled to accommodate the units of each factor, there is no interruption in the center of the design space. In Eqs. (4) and (25) a positive value indicates the effect that causes the optimization, while a negative value represents the inverse relationship between the responses and factors. The quadratic model is accepted as it is selected by the software for the response. The *R*^*2*^ (coefficient of determination) and Adj-*R*^*2*^ (adjusted-*R*^*2*^) models are 0.9822 and 0.9795, respectively. This value showed a good fit between the modeled value and the experimental data point (Fig. 3A and B). Also, this means that 98.22% of the changes in $N_{{{\text{CO}}_{2} }}$ are explained by the independent variables.

As shown in Fig. 3A and B, the residuals are placed on a straight line and are normally distributed. The predicted and actual values for all responses were close to each other. This observation indicates that these models are appropriate for the experimental data and can be used to analyze and predict the $N_{{{\text{CO}}_{2} }}$ performance.

The least-squares residuals are a powerful tool for checking the models’ adequacy. The hypothesis of constant variance at specific levels in Fig. 3C and D was considered by plotting the residual obtained from the model versus the predicted response values. There is a random distribution of points above and below the x-axis between − 3.8419 and + 3.8419 and − 3 to + 3 for externally and internally studentized residuals, respectively, as shown in Fig. 3C and D. This conclusion examines the adequacy and reliability of the models, and a constant variance is observed via the response amplitude. As an additional tool to check the adequacy of the final model, the normal probability diagram of the studentized residuals is represented in Fig. 3B. If the model is appropriate, the points on the normal probability graph versus the residuals should form a straight line. In these graphs, the points follow a straight line and confirm that the errors are typically distributed with a constant and zero but indeterminate variance as the primary hypothesis of the studies. Thus, all graphs were shown to be desirable, and there was no reason to reject the results. ANOVA and the statistical parameters for the CO_2_ mass transfer flux from the CCD are introduced in Table 3. The model fit analysis was performed using an ANOVA test and a lack of fit. An experiment's data will fit well with the model if it has significant regression and does not show a significant lack of fit. The statistical significance was assessed using the factors, interactions, and *p*-value of the model.

As can be seen from Table 3, the predicted *R*^*2*^ of 0.9740 is in reasonable agreement with the adjusted-*R*^*2*^ of 0.9795; i.e., the difference is less than 0.2. *Adeq* precision measures the signal-to-noise ratio. A ratio greater than 4 is desirable. The ratio of 135.873 indicates an adequate signal. This model can be used to navigate the design space. The model *p*-value for CO_2_ mass transfer flux is smaller than 0.0001, which indicates that the model parameters are significant. The *P*-values less than 0.050 indicate that model terms are significant. The values greater than 0.100 indicate the model terms are not nominal. If there are many insignificant model terms (not counting those required to support hierarchy), model reduction may improve the model. Also, the model F-value of 360.43 implies the model is significant. There is only a 0.01% chance that an F-value this large could occur due to noise.

#### Interaction of factors

**Figure 4:**
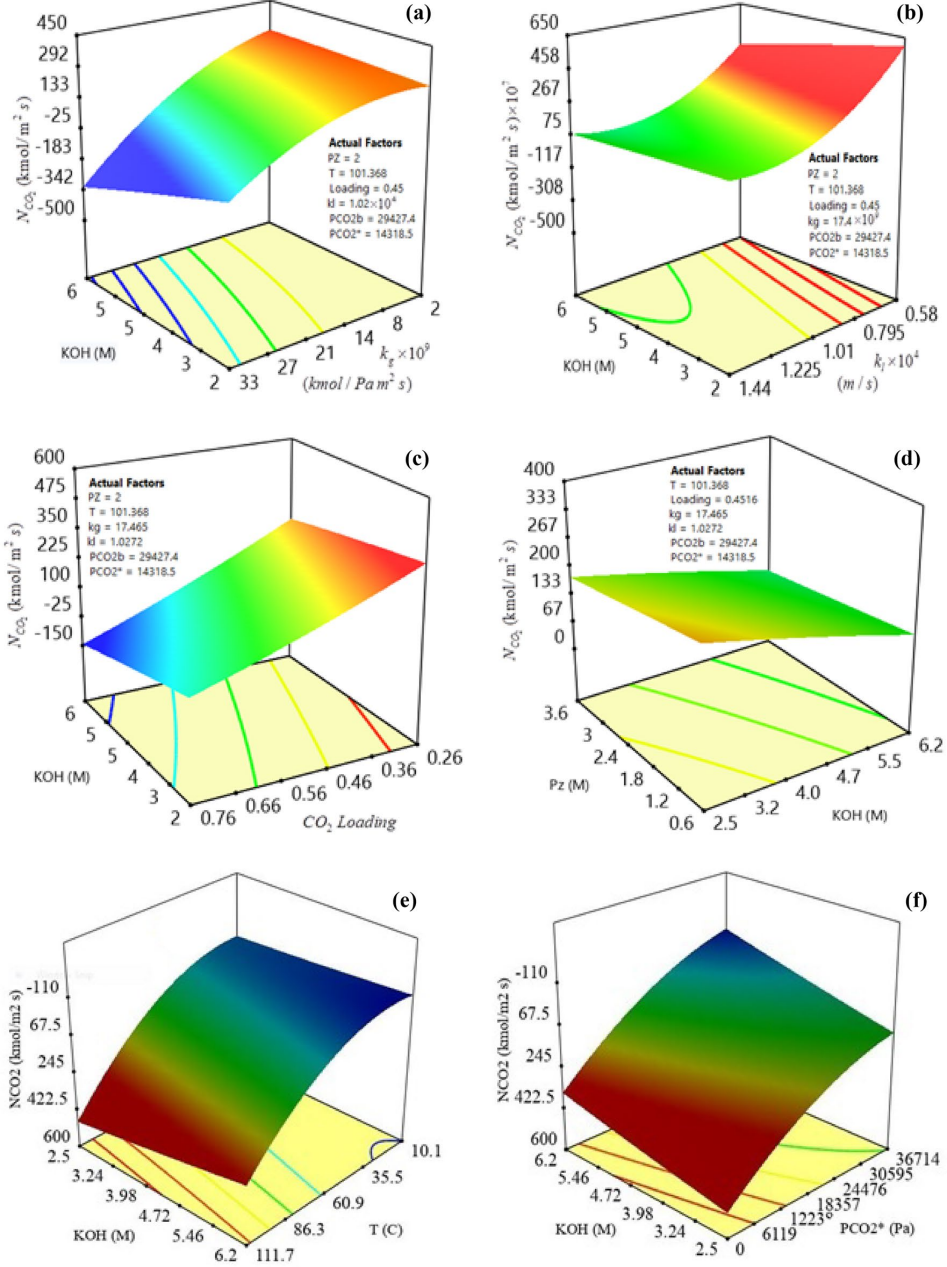
Response surfaces plots of CO_2_ transfer flux variation with KOH concentration as a function of; (**a**) gas film side mass transfer coefficient, (**b**) liquid film side mass transfer coefficient, (**c**) CO_2_ loading, (**d**) Pz concentration, (**e**) temperature, and (**f**) equilibrium CO_2_ partial pressure.

In this study, version 11.0 of Design-Expert was used to represent three-dimensional (3-D) response surfaces and segmentation of the data set in supplementary Table S1. The 3-D response surface curves for mutual understanding of variable parameters (i.e., KOH and Pz concentrations, temperature, CO_2_ loading, gas film, and liquid side mass transfer coefficients, the gas bulk partial pressure of CO_2_, and equilibrium CO_2_ partial pressure), and determining the best level of each variable for maximum response in CO_2_ mass transfer flux were plotted in Fig. 4. Also, in these figures, the graphs were used to identify the best range of all eight variable factors. The label lines on the scheme and the different colors of the graphs and response surfaces represented the types of degrees of interaction based on the CO_2_ mass transfer flux (see Eqs. (24), and (25)).

The interaction of the two variable parameters (KOH concentration and equilibrium CO_2_ partial pressure) on the mass transfer flux was significant compared to the other six parameters shown in Fig. 4. The interaction effects of the KOH and Pz concentrations, CO_2_ loading, gas film mass transfer coefficient, liquid side mass transfer coefficient, and equilibrium CO_2_ partial pressure were similar. However, the slope of the curve at the three-dimensional response surface (see Fig. 4b and f) showed that the *k*_*l*_ and $p_{{{\text{CO}}_{2} }}^{*}$ a higher effect on the mass transfer flux compared to the other parameters level of setup. In conclusion, these two parameters played an essential role in influencing the mass transfer flux, which was consistent with the results obtained from the regression model ANOVA. The effect of these two parameters on the mass transfer flux comes from the number of CO_2_ molecules in the liquid bulk, which causes an enhancement in the reaction between the free Pz and CO_2_ molecules and leads to an increase in the mass transfer flux. In contrast, according to the RSM, the optimum value of mass transfer flux decreased (blue area in Fig. 4e) when the temperature and the bulk CO_2_ partial pressure increased to the given level, which suggested that reaction rate is the principal parameter in controlling the CO_2_ absorption, too. As seen from the 3-D response surface in Fig. 4a, c, and d, the $N_{{{\text{CO}}_{2} }}$ was modified with increases in the Pz concentration, CO_2_ loading, and gas film side mass transfer coefficient. It was apparent from the figures that there was weak interaction between the gas bulk partial pressure and another parameter. In contrast, the importance of the interaction between the gas film side mass transfer coefficient and the other parameters was great.

#### Process optimization

One goal of this study is to observe independent variables (i.e., C_KOH_, C_Pz_, T, CO_2_ loading, k_g_, k_l_, $p_{{{\text{CO}}_{2} ,b}}$, and $p_{{{\text{CO}}_{2} }}^{*}$) in some way to obtain maximum mass transfer flux performance. According to the experiments performed, the optimization of the response surface method has proposed various combinations of variables to obtain a mass transfer flux performance of more than 95%. In this method, an optimization run with 48.6 kmol/m^2^.s of CO_2_ mass transfer flux was chosen. This optimal point can be acquired within the following limitations, as shown in Table 4.

**Table 4:** Optimization of the CO_2_ absorption by RSM-CCD.

Parameter and response	Constrain	Low	High	Optimum condition
C_KOH_ (mol/l)	In range	2.5	6.2	5.23
C_Pz_ (mol/l)	In range	0.6	3.6	3.034
T (C)	In range	10.1	111.7	51.18
CO_2_ loading (−)	In range	0.262	0.761	0.479
K_g_ (kmol/m^2^ s Pa)	In range	1.63	33.3	12.59
k_l_ (m/s)	In range	0.58	1.44	0.6787
$p_{{{\text{CO}}_{2} ,b}}$(Pa)	In range	0.00	60,056	54,348.9
$p_{{{\text{CO}}_{2} }}^{*}$(Pa)	In range	0.0	36,714	3887.1
$N_{{{\text{CO}}_{2} }}$(kmol/ m^2^.s)	Maximize	0.00636	0.01599	48.6

Using numerical optimization, you can select the desired value for each input and response parameter. Here, the possible input optimizations that can be selected include the range, maximum, minimum, target, none (for responses), and adjust to specify an optimized response value for a certain set of specified conditions. In this study, the input data variables were given to determine the range value of the response to obtain the maximum.

**Figure 5:**
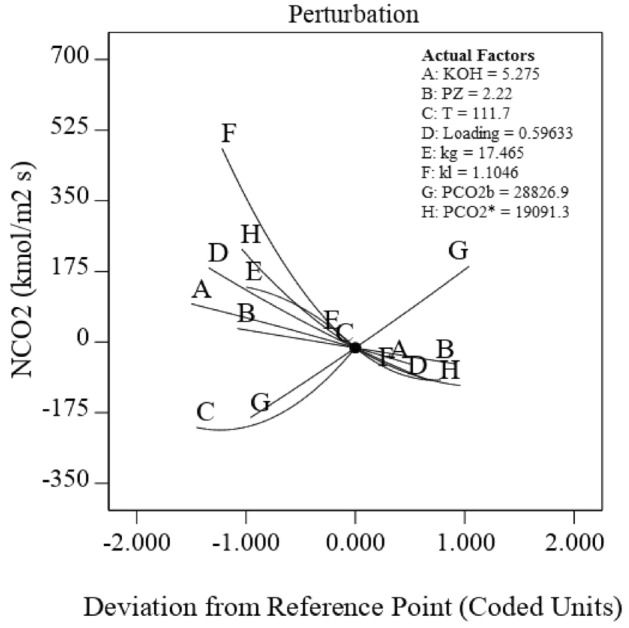
Deviation curves for CO_2_ mass transfer flux with coded factors.

The deviation diagram shows the overall effect of all parameters on the response performance, and the center point (0) is the operating midpoint range. The perturbation scheme for comparing the effects of all eight operating parameters (KOH and Pz concentrations, temperature, CO_2_ loading, gas film, liquid side mass transfer coefficient, gaseous bulk partial pressure of CO_2_, and equilibrium CO_2_ partial pressure of the bulk solution) at the reference point is shown in Fig. 5. It was observed from Fig. 5 that the CO_2_ mass transfer flux increases with the decrease of KOH concentration (A), Pz concentration (B), CO_2_ loading (D), gas film mass transfer coefficient (E), liquid side mass transfer coefficient (F), and the equilibrium CO_2_ partial pressure of the bulk solution (H). Nevertheless, the reduction rate of response was further for k_l_ and $p_{{CO_{2} }}^{*}$ in comparison to the four above-mentioned parameters (KOH concentration, Pz concentration, *k*_*g*_, and *k*_*l*_). It is also clear that the $N_{{{\text{CO}}_{2} }}$ increases with an increase in the temperature (C) and $p_{{{\text{CO}}_{2} ,b}}$ (G) due to increase CO_2_ agents in the interface and then in the solution.

### ANN approach

#### Back-propagation algorithm for the absorption process

To find the optimal method for the ANN, study used three different learning algorithms, including the MLP Backpropagation (BP) algorithms, i.e., Levenberg–Marquardt (LM)^103^, Bayesian Regularization (BR)^104^, and scaled Conjugate Gradient (SCG)^105^. After preparation of the data gathering from the experimental work, the solution concentration of KOH and Pz, temperature, CO_2_ loadings, gas side mass transfer coefficient (*k*_*g*_), liquid film mass transfer coefficient (*k*_*l*_), the partial pressure of CO_2_ at the gas–liquid interface ($p_{{{\text{CO}}_{2} ,b}}$) and the equilibrium partial pressure of CO_2_ in bulk ($p_{{{\text{CO}}_{2} }}^{*}$) as input data and absorption flux of a gas into a liquid ($N_{{{\text{CO}}_{2} }}$) as output data. Training, validation, and test datasets were created using the CO_2_ absorption data. The network chooses all of the input data at random. Network training accounted for 70% of total data points (237 datasets), whereas network validation and testing accounted for 30% of total data points (15%) (102 datasets). To approximate the network weights and biases, the training dataset was used, and to distinguish the predictability of the developed network, the test and validation datasets were used. In the next step, the input and output dataset variables in the range of − 1 and 1 were normalized based on Eq. (26).

26 $$X_{{{\text{norm}}}} = 2\frac{{(X - X_{\min } )}}{{(X_{\max } - X_{\min } )}} - 1$$

where, $X_{{{\text{norm}}}}$, $X$,$X_{\max }$ and, $X_{\min }$ are normalized data, raw input variable, maximum and minimum of the dataset, respectively. To identify the appropriate quantity of network parameters during network training, the predicted network error must be kept to a minimal at each step of the MSE in each iteration. We applied the same criteria for this aim, such as the MSE and the square of the coefficient of correlation (*R*^*2*^). The following are the mathematical equations for the functions given^102,106^:

27 $${\text{MSE}} = \frac{1}{n}\sum\limits_{i = 1}^{n} {\left( {Y_{{{\text{predicted}}}} - Y_{{{\text{actual}}}} } \right)^{2} }$$

28 $$R^{2} = \frac{{\sum\limits_{i = 1}^{n} {\left( {Y_{{{\text{predicted}}}} - Y_{{{\text{actual}}}} } \right)^{2} } }}{{\sum\limits_{i = 1}^{n} {\left( {Y_{{{\text{predicted}}}} - Y_{{{\text{mean}}}} } \right)^{2} } }}$$

where, $Y_{{{\text{actual}}}}$ is experimental data and $Y_{{{\text{predicted}}}}$ is the network predicted data.

#### Optimization of MLP and RBF models

In this research, different neuron activation functions were used, and eventually, sigmoid, and the *pure-line* transfer functions for hidden and output layers were chosen, respectively. The mathematical functions were applied as activation functions for all neurons of the covered layers that are given in Table 5.

**Table 5:** Mathematical and graph symbols of activation functions.

$${\text{Graph and Symbol function}}$$		
${\text{Mathematical algorithm function}}$	$a = purelin(n) = n$	$a = \tan sig(n) = \frac{2}{{(1 + \exp ( - 2*n)^{ - 1} )}}$

**Figure 6:**
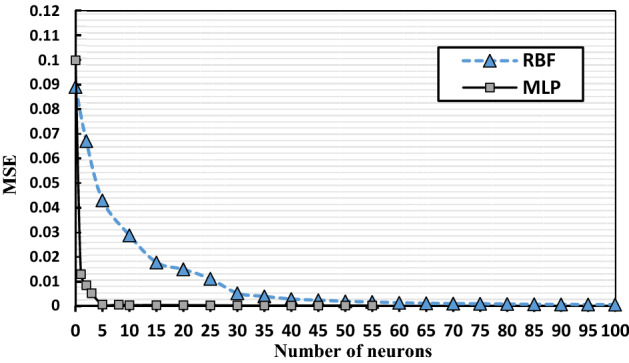
Optimized number of neurons for RBF and MLP models structures.

During the training phase, the ideal number of neurons was chosen based on the minimal MSE and *R*^*2*^. As shown in Fig. 6, the number of hidden neurons in the MLP model was changed from 1 to 55, with the ideal number of neurons determined by the MSE minimum value. Hidden layer activation functions should add nonlinearity to analyze network reliability. For the comparison research, thirty-one neurons were investigated, and the BR algorithm was chosen because it had the lowest MSE of 1.910^–4^ and the highest regression value (*R*^*2*^) of 0.9971, as shown in Table 6.

**Table 6:** MLP training algorithms for the absorption process.

Algorithms	Function	MSE	R^2^	Epoch	Best linear equation
${\text{Levenberg - Marquardt }}\;{\text{backpropagation}}$	$trainlm$	0.00191	0.9858	10	Output = 0.98*Target − 0.0065
${\text{Bayesian Regularization}}$(${\text{BR}}$)	$trainbr$	0.00019	0.9971	100	Output = 1*Target + 0.0024
$${\text{Scaled Conjugate}}$$$${\text{Gradient}}$$ ($${\text{SCG}}$$)	$$trainscg$$	0.00282	0.9808	15	Output = 0.98*Target − 0.0013

**Figure 7:**
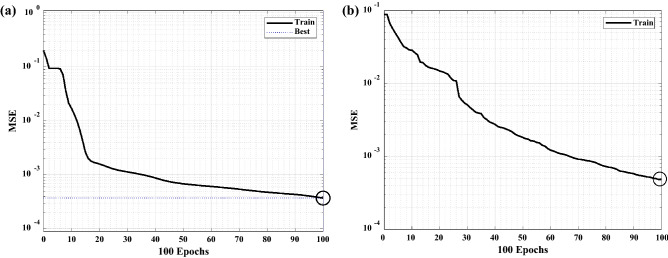
Comparison MSE of MLP (**a**) and RBF (**b**) models performance for the absorption process.

The ideal number of neurons for the RBF model with Gaussian functions at the single layer was 100, as shown in Fig. 6. MLP and RBF model networks had excellent MSE validation performances of 0.00019 and 0.00048 at 100 epochs, respectively, as shown in Fig. 7.

Supplementary Figure S4 depicts a basic overview of MLP and RBF structure approaches. The first layer in MLP structure is the input layer, which receives data (i.e., solution concentration of KOH and piperazine (Pz), temperature, CO_2_ loadings, kmol/Pa m^2^ s, liquid film mass transfer coefficient (*k*_*l*_), the partial pressure of CO_2_ at the gas–liquid interface ($p_{{{\text{CO}}_{2} ,b}}$) and the equilibrium partial pressure of CO_2_ in bulk ($p_{{{\text{CO}}_{2} }}^{*}$) as input parameters data) is imported into the network. The last layer is the output one, i.e., absorption flux of a gas into a liquid ($N_{{{\text{CO}}_{2} }}$) as output data, which gives the target data. Regarding the optimization process for the neurons in the MLP structure, the number of neurons at the first and second hidden layers were twenty-five and five, respectively. Furthermore, the number of neurons in the output layer with the pure-linear (linear) transfer function had to be one.

**Figure 8:**
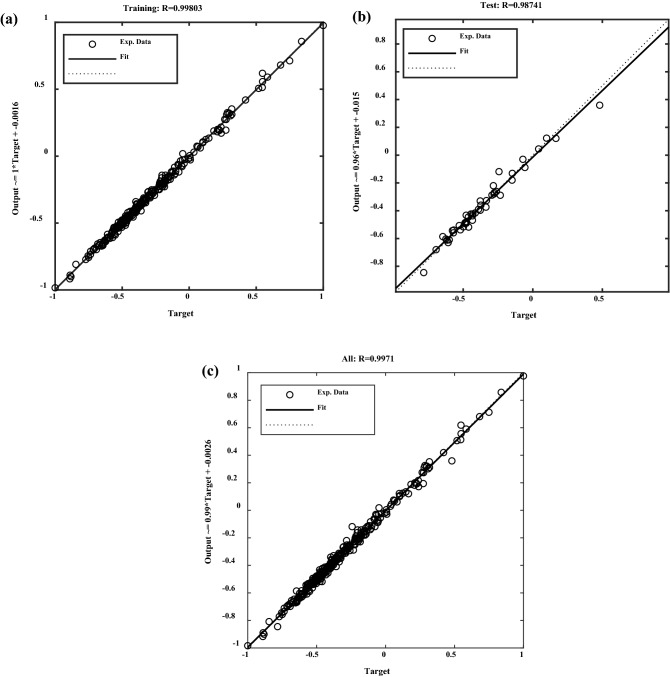
MLP model regression plots prediction (**a**) during training; (**b**) during the test, and (**c**) all the data.

Figure 8 shows the close relationship between the MLP model of ANN outputs and our target values during the network training and testing of CO_2_ absorption process data. In the network training and testing of the CO_2_ absorption process data, Figure 8 illustrates the strong correlation between the MLP model outputs and our objective values. The values for the MLP model displayed in Figure 8 (i.e., the training, validation, test, and all the data) were almost one (*R*^2^= 0.9971). Further confirmation of the close resemblance between the anticipated absorption values from the CO2 absorbed dataset and the MLP outputs is provided in Figure 8.

**Figure 9:**
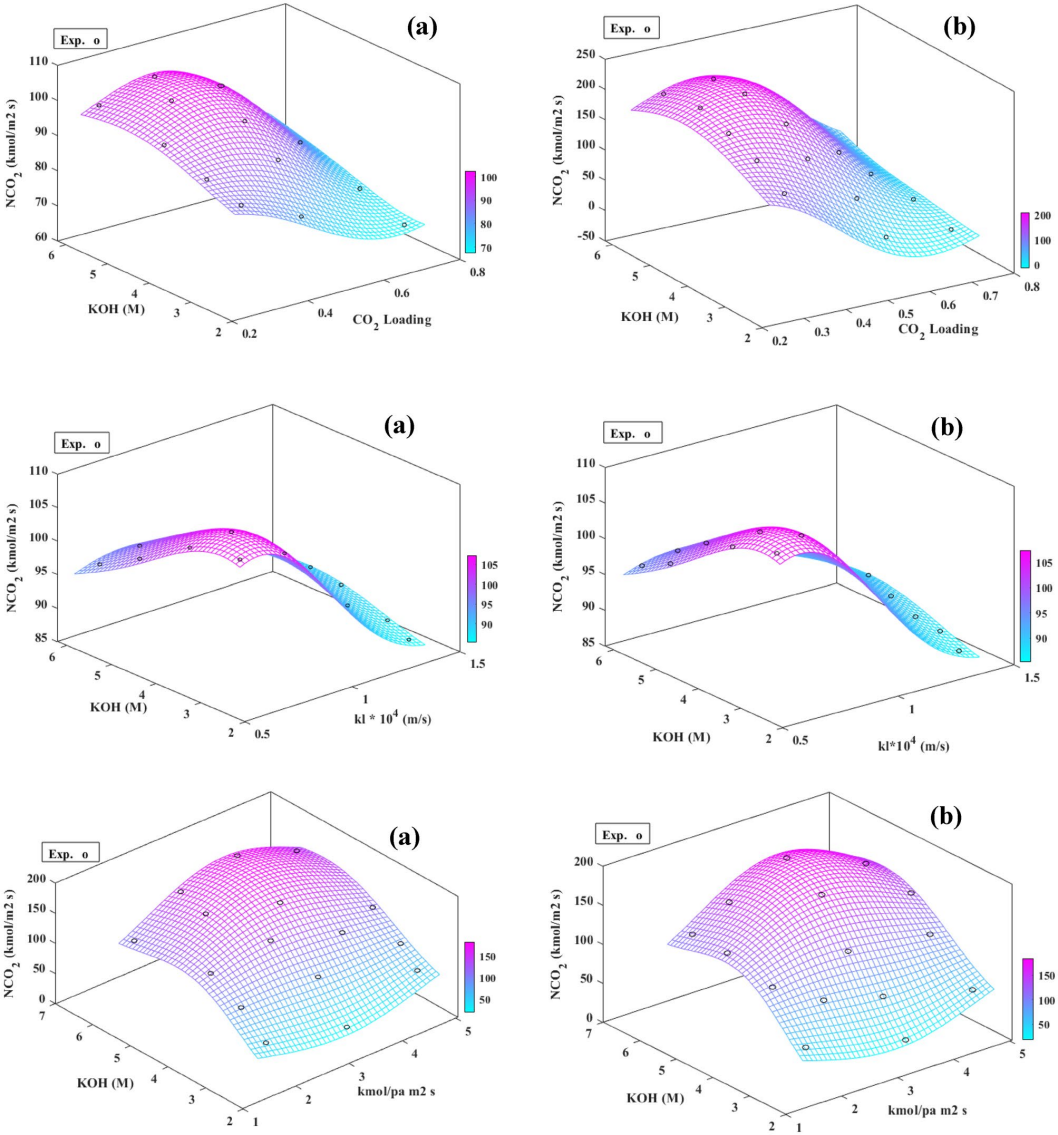

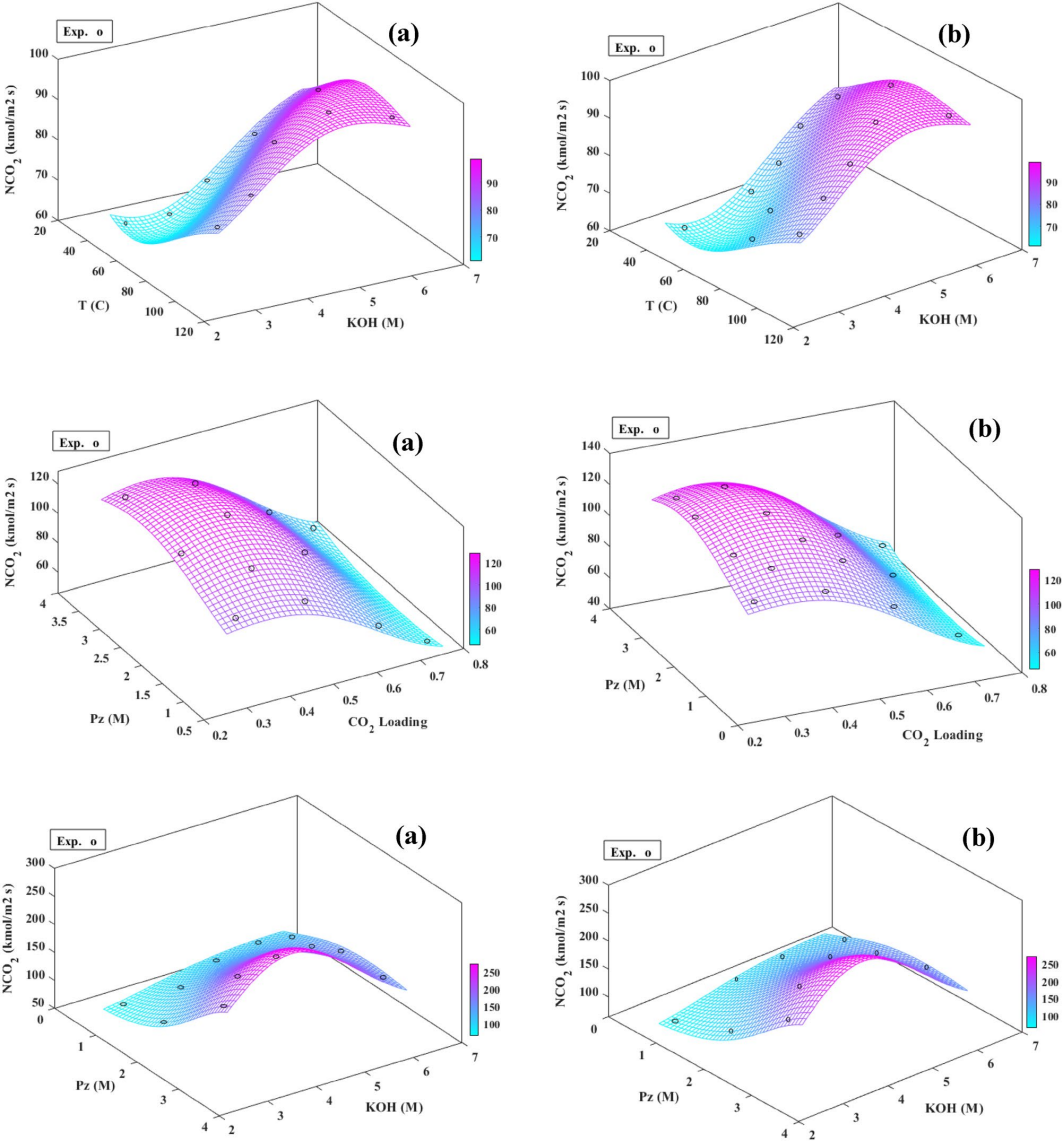
Response surfaces plot artificial neural networks of the MLP (**a**) and RBF (**b**) models for the prediction of flux absorption (*N*_CO2_).

**Figure 10:**
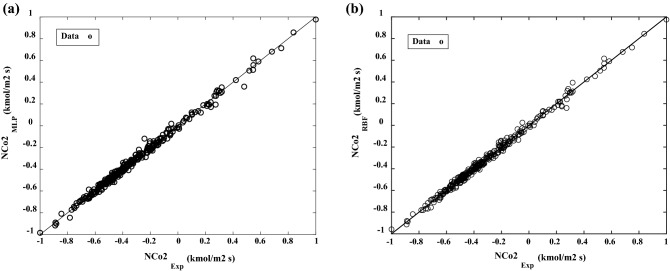
MLP (**a**) and RBF (**b**) normalized models fitting for experimental and predicted absorption flux (*N*_CO2_).

The 3-D curves of a response surface for both algorithms, as shown in Fig. 9, were drawn to understand the interaction of the variable parameters (i.e., solution concentration of KOH and piperazine (Pz), temperature, CO_2_ loadings, kmol/Pa.m^2^.s, liquid film mass transfer coefficient (*k*_*l*_), the partial pressure of CO_2_ at the gas–liquid interface ($p_{{{\text{CO}}_{2} ,b}}$) and the equilibrium partial pressure of CO_2_ in bulk ($p_{{{\text{CO}}_{2} }}^{*}$) to locate each variable response of the absorption process. Furthermore, in these figures, the plots were applied to detect the CO_2_ absorption range of each of the two variables. The predicted and experimentally normalized data of the MLP and RBF models are fitted in Fig. 10 for the absorption process. These results show that the predicted models are well matched with the experimental flux absorption data. Table 7 (structure 25 and 5 neurons for the first and second hidden layers, MSE = 0.00019 and, *R*^*2*^ = 0.9971 for all data) shows the weights (*w*) and biases (*b)* obtained by the best ANN-MLP model. Using the data in Table 7 and the specified ANN-MLP structure provided in the ANN section, the best ANN-MLP model discussed in this work can be completely recreated. The findings of this study could be added to the database of CO_2_ absorption and used to forecast gas absorption behaviors using the suggested prediction matrix. A reliable and effective hybrid strategy for simulating CO_2_ absorption under various process circumstances has been proposed in this study. The ANN algorithm could be modified and improved by adding new data, has a fast prediction speed, and is based on experimental data. When building different factors, the observed CO_2_ absorption at various process circumstances is necessary, but the ANN structure does not use the absorption model parameters as an input to anticipate the absorption parameters. The interaction of variables for nonlinear problems that are difficult for numerical methods to capture could be discovered using experimental results, and ANN has excellent training and adaptation functionality.

**Table 7:** Weight matrix using the MLP-ANN model output developed.

Neuron		1	2	3	4	5	6	7	8	9	10	11	12	13
First hidden layer	*w*_*i*_	− 0.0588	− 0.0201	0.0462	− 0.0803	0.0833	− 0.0546	− 0.0526	− 0.0252	− 0.0090	0.1532	− 0.2422	0.1279	0.0141
		− 0.0545	0.0217	0.0419	− 0.0401	0.0652	− 0.0456	− 0.0402	0.0190	0.2381	0.0485	− 0.0269	0.6724	− 0.0479
		− 0.0836	− 0.0712	− 0.0680	− 0.0140	0.0799	− 0.0946	− 0.0897	− 0.0823	− 0.3736	− 0.0997	0.1757	0.1861	0.1000
		0.0489	0.0408	0.1397	− 0.0314	− 0.0310	0.0563	0.0530	0.0457	− 0.5236	0.2058	0.4780	− 0.7155	− 0.0504
		0.0919	0.0409	− 0.0677	− 0.0011	− 0.0330	0.0983	0.0927	0.0501	0.3585	− 0.0786	− 0.3345	0.4551	− 0.0333
		0.1152	0.0357	0.1870	0.1328	− 0.1379	0.1142	0.1071	0.0446	0.0058	0.1887	0.0237	0.6284	− 0.0139
		0.0672	0.0136	0.1872	0.1638	− 0.1200	0.0523	0.0523	0.0136	0.7614	0.1898	1.3287	0.5551	0.0215
		− 0.0535	− 0.0295	0.1120	0.0844	− 0.0020	− 0.0605	− 0.0557	− 0.0372	0.1601	0.0984	0.2171	0.0462	0.0463
		–	–	–	–	–	–	–	–	–	–	–	–	–
		–	–	–	–	–	–	–	–	–	–	–	–	–
		–	–	–	–	–	–	–	–	–	–	–	–	–
		–	–	–	–	–	–	–	–	–	–	–	–	–
		–	–	–	–	–	–	–	–	–	–	–	–	–
		–	–	–	–	–	–	–	–	–	–	–	–	–
		–	–	–	–	–	–	–	–	–	–	–	–	–
	*b*	0.1581	0.1581	0.1581	0.1581	0.1581	0.1581	0.1581	0.1581	0.1581	0.1581	0.1581	0.1581	0.1581
Second hidden layer	*w*_*i*_	0.0583	0.9003	0.1429	0.5379	0.0045	–	–	–	–	–	–	–	–
		− 0.3804	− 0.9897	− 0.2525	− 0.4116	− 0.2927	–	–	–	–	–	–	–	–
		− 0.2125	− 0.2026	0.1630	0.3131	− 0.0844	–	–	–	–	–	–	–	–
		0.1982	− 0.0668	− 0.0255	− 0.0868	0.1066	–	–	–	–	–	–	–	–
		0.1028	0.4943	0.0461	− 0.2821	− 0.6051	–	–	–	–	–	–	–	–
		0.0023	0.0169	0.0030	− 0.0297	0.0707	–	–	–	–	–	–	–	–
		0.5424	0.7820	− 0.1236	− 0.0777	− 0.1832	–	–	–	–	–	–	–	–
		− 0.1061	0.9620	0.4573	0.0128	− 0.5454	–	–	–	–	–	–	–	–
		− 0.0840	− 0.0645	− 0.0647	0.1429	0.9620	–	–	–	–	–	–	–	–
		− 0.2119	− 0.3133	− 0.3871	0.3073	− 0.1114	–	–	–	–	–	–	–	–
		0.0324	− 0.0048	0.0021	− 0.0597	0.2101	–	–	–	–	–	–	–	–
		− 0.2451	− 0.0114	0.0889	0.2130	0.0162	–	–	–	–	–	–	–	–
		–	–	–	–	–	–	–	–	–	–	–	–	–
		–	–	–	–	–	–	–	–	–	–	–	–	–
		–	–	–	–	–	–	–	–	–	–	–	–	–
	*b*	− 0.3771	− 0.3771	− 0.3771	− 0.3771	− 0.3771	–	–	–	–	–	–	–	–
Output layer	*w*_*l*_	− 0.7715	− 1.6764	0.8165	1.0627	0.8821								
	*b*	0.0212	–	–	–	–	–	–	–	–	–	–	–	–

Neuron		14	15	16	17	18	19	20	21	22	23	24	25	
First hidden layer	*w*_*i*_	− 0.3403	− 0.8755	0.4217	0.0558	0.9511	0.0017	− 0.6651	− 0.3656	− 0.3916	0.0283	0.0295	− 0.0143	
		− 0.3996	− 0.9866	− 0.0355	0.0477	− 0.1449	− 0.0339	0.2378	− 0.2060	− 0.1476	0.4997	− 0.0631	− 0.0082	
		0.0402	− 0.5170	− 0.2262	0.0812	− 0.4453	0.0531	0.7564	− 0.7238	0.7645	− 0.2237	0.1515	− 0.0872	
		0.2614	0.4433	0.2902	− 0.0475	− 0.0677	− 0.0297	− 0.0889	0.0970	0.1906	− 0.1269	− 0.0624	0.1049	
		0.1479	− 0.4646	− 0.0585	− 0.0889	0.4950	− 0.0133	− 0.6122	0.0952	− 0.4722	0.0654	− 0.0439	− 0.0295	
		− 0.2506	0.7261	0.1371	− 0.1088	− 0.0163	− 0.0012	0.0778	0.3751	− 0.3595	0.0878	− 0.0121	0.1693	
		− 0.0118	1.0513	0.1995	− 0.0640	− 0.9281	0.0137	− 0.2912	− 0.4742	0.3659	0.3542	0.0514	0.1497	
		0.6229	− 0.5241	0.0736	0.0507	− 0.0463	0.0207	0.6417	0.3335	− 0.1884	− 0.0125	0.0841	0.0643	
		–	–	–	–	–	–	–	–	–	–	–	–	
		–	–	–	–	–	–	–	–	–	–	–	–	
		–	–	–	–	–	–	–	–	–	–	–	–	
		–	–	–	–	–	–	–	–	–	–	–	–	
		–	–	–	–	–	–	–	–	–	–	–	–	
		–	–	–	–	–	–	–	–	–	–	–	–	
		–	–	–	–	–	–	–	–	–	–	–	–	
	*b*	0.1581	0.1581	0.1581	0.1581	0.1581	0.1581	0.1581	0.1581	0.1581	0.1581	0.1581	0.1581	
Second hidden layer	*w*_*i*_	–	–	–	–	–	–	–	–	–	–	–	–	
		–	–	–	–	–	–	–	–	–	–	–	–	
		–	–	–	–	–	–	–	–	–	–	–	–	
		–	–	–	–	–	–	–	–	–	–	–	–	
		–	–	–	–	–	–	–	–	–	–	–	–	
		–	–	–	–	–	–	–	–	–	–	–	–	
		–	–	–	–	–	–	–	–	–	–	–	–	
		–	–	–	–	–	–	–	–	–	–	–	–	
		–	–	–	–	–	–	–	–	–	–	–	–	
		–	–	–	–	–	–	–	–	–	–	–	–	
		–	–	–	–	–	–	–	–	–	–	–	–	
		–	–	–	–	–	–	–	–	–	–	–	–	
		–	–	–	–	–	–	–	–	–	–	–	–	
		–	–	–	–	–	–	–	–	–	–	–	–	
		–	–	–	–	–	–	–	–	–	–	–	–	
	*b*	–	–	–	–	–	–	–	–	–	–	–	–	
Output layer	*w*_*l*_	–	–	–	–	–	–	–	–	–	–	–	–	
	*b*	–	–	–	–	–	–	–	–	–	–	–	–	

*w*_*i*_*:* weights between input and hidden layers.

*w*_*l*_: weights between hidden and output layers.

## Conclusion

Growing requests for both analysis and process optimization combined with the increasing availability of statistical software and growing computing power have led to the widespread use of RSM and ANN modeling tools. In the RSM approach, the impact of multiple parameters on the *N*_CO2_ has been studied. We optimized the Pz-KOH-CO_2_ system (in the CO_2_ removal process) to maximize the mass transfer flux using simulation/optimization RSM. The RSM with CCD was applied for the development of the suitable model using the least-squares procedure. The deviation errors were acquired for all absorption functions lower than 0.0001, and the results were as follows: (i) The *R*^*2*^ and *Adj-R*^*2*^ models were obtained at 0.9822 and 0.9795, respectively, and this value showed a good fit between the modeled value and the experimental data point. Therefore, it was found that the models were successfully examined and all verified with experimental data, and specified the values of the optimum variables to maximize absorption functions. (ii) The model *p*-value was less than 0.0001, which indicates that the model parameters are significant. Furthermore, the model F-value was 360, which indicates only a less than 0.01% possibility that this high value of F-value may be due to noise. (iii) The optimization of the response surface method has proposed various combinations of variables to obtain a mass transfer flux performance of more than 95%. In this method, an optimization run with 48.6 kmol/m^2^ s of CO_2_ mass transfer flux was obtained. This optimal absorption condition was acquired at Pz and KOH concentration of 1.31 and 4.34, respectively, temperature of 22.28 C, CO_2_ loading of 0.398, gas film and liquid side mass transfer coefficient of 7.68 kmol/Pa m^2^ s and 1.046 m/s, respectively, bulk partial pressure of 45,862.2 Pa, and an equilibrium partial pressure of 10,080.2 Pa. The experimental yield of $N_{{{\text{CO}}_{2} }}$ was 699 kmol/m^2^ s.

In the ANN approach, the operating parameters for the prediction of the mass transfer flux were developed through artificial neural networks. The BR back-propagation algorithm has the best performance among other MLP models with a tangent sigmoid transfer function (trainer) as hidden layers and a linear transfer function (*pure*-*line*) as the output layer. The best integrated MLP model for the CO_2_ absorption flux process gives an overall *R*^*2*^ value of 0.9971 with an MSE value of 0.00019 at 100 epochs. The optimal number of neurons for another algorithm (RBF) model was 100 at the single layer, with the bet MSE validation performance network of 0.00048 at 100 epochs. A comparison of MSE and performance values ​​showed little difference between the results of both RBF methods, but the MLP model with fewer neurons provided more accuracy. By utilizing ANN algorithms, we have the possibility of significantly reducing the amount of time spent on experimental tasks. The ML algorithms could then be updated whenever a new dataset becomes available because they would have been trained using the previously collected experimental data. This ML technique could also be employed to build a substantial numerical frame to improve the CO_2_ absorption process by utilizing process conditions. We created models that can reliably and accurately reproduce the experimental data on CO_2_ absorption. The present study's findings were used to construct the ANN matrix, which would be improved in the future to forecast CO_2_ absorption. The current algorithm proposed is not strictly specific to the general flux absorption of CO_2_, but can be used for absorption. The ANN development based on different materials with extended various conditions can be added in future studies.

## Supplementary Information

Supplementary Information 1.

Supplementary Information 2.

## Data Availability

The datasets used and analyzed during the current study are available from the corresponding author upon reasonable request. If you need to find out about the ANN structure of the simulation codes, you can contact the following email: h.mashhadimoslem@gmail.com.

## List of symbols

b: Bias (−)
C_i_: Center points (−)
F_k_: Nonlinear activation transfer functions
f: Function
G: Gaussian function
g: Output vector (−)
i: Number of neurons in hidden layer
k: Position vector
K_g_: Gas phase mass transfer coefficient (kmol/m^2^ s Pa)
K_l_: Liquid side mass transfer coefficient (m/s)
n: Number of neuron
N: Number of data set for training (−)
$N_{{{\text{CO}}_{2} }}$: Mass transfer flux (kmol/m^2^ s)
$p_{{{\text{CO}}_{2} ,b}}$: Gas bulk partial pressure of CO_2_ (Pa)
Pz: Piperazine (−)
$p_{{{\text{CO}}_{2} }}^{*}$: Equilibrium CO_2_ partial pressure of the bulk solution (Pa)
$p_{{{\text{CO}}_{2} ,i}}$: Interface partial pressure of CO_2_ (Pa)
$p_{{{\text{CO}}_{2} }}^{*}$: Equilibrium CO_2_ partial pressure of the bulk solution (Pa)
$p_{{{\text{CO}}_{2} ,i}}$: Interface partial pressure of CO_2_ (Pa)
R^2^: Correlation coefficient
s: Direction vector
T: Temperature (°C)
W: Weight factor (−)
W_ij_: Weight related to each hidden neuron (−)
X_Exp_: Experimental value (−)
${\text{Q}}_{{{\text{CO}}_{2} }}$: CO_2_ flow rate, l/min
X_Sim_: Simulation value (−)
x: Input variable (−)
x_i_: Input examples (attributes)
y_j_: Target output

## Greek letters

α: Constants in turbulence models (−)
$\beta_{jk}$: Bias weight for neuron j in layer k
$\gamma_{jk}$: Neuron j’s output from k’s layer
θ: Threshold limit (−)
$\upsigma$: Width of radial basis function (RBF) kernel (−)
σ_i_: Spread of Gaussian function (−)

## Abbreviations

AARE: Average absolute relative error (%)
ANN: Artificial Neural Networks
MSE: Mean Square Error

## Terminology

Neurons: Neurons are the basic units of the large neural network
Bias: Bias is a constant which helps the model in a way that it can fit best for the given data
Activation function: The activation function is a mathematical function in between the input feeding the current neuron and its output going to the next layer
Weight: Represents the importance and strengths of the feature/input to the Neurons
Epoch: In the training process, the inputs enter each training step and give output that are compared with the target to calculate an error. With this process, weights and biases are calculated and modified in each epoch

## References

[1] Lu J-G (2015). Prediction and validation of physical property for a CO_2_ capture agent of aqueous (potassium citrate+ 2-amino-2-methyl-1-propanol). J. Nat. Gas Sci. Eng..

[2] Masoumi S, Keshavarz P, Rastgoo Z (2014). Theoretical investigation on CO_2_ absorption into DEAB solution using hollow fiber membrane contactors. J. Nat. Gas Sci. Eng..

[3] Shamiri A (2016). Absorption of CO_2_ into aqueous mixtures of glycerol and monoethanolamine. J. Nat. Gas Sci. Eng..

[4] Pashaei H, Zarandi MN, Ghaemi A (2017). Experimental study and modeling of CO_2_ absorption into diethanolamine solutions using stirrer bubble column. Chem. Eng. Res. Des..

[5] Pashaei H, Ghaemi A, Nasiri M (2016). Modeling and experimental study on the solubility and mass transfer of CO_2_ into aqueous DEA solution using a stirrer bubble column. RSC Adv..

[6] Pashaei, H., Mirzaei, F., & Ghaemi, A. Experimental study and modeling of mass transfer flux of CO_2_ absorption with amine solution in bubble column. *J. Chem. Pet. Eng.* (2022).

[7] Houshmand A (2013). Anchoring a halogenated amine on the surface of a microporous activated carbon for carbon dioxide capture. J. Taiwan Inst. Chem. Eng..

[8] Shafeeyan MS (2015). Modeling of carbon dioxide adsorption onto ammonia-modified activated carbon: Kinetic analysis and breakthrough behavior. Energy Fuels.

[9] Pashaei H, Ghaemi A (2020). CO2 absorption into aqueous diethanolamine solution with nano heavy metal oxide particles using stirrer bubble column: Hydrodynamics and mass transfer. J. Environ. Chem. Eng..

[10] Rezaei B, Riahi S (2016). Prediction of CO_2_ loading of amines in carbon capture process using membrane contactors: A molecular modeling. J. Nat. Gas Sci. Eng..

[11] Heydarifard M (2018). Reactive absorption of CO_2_ into Piperazine aqueous solution in a stirrer bubble column: Modeling and experimental. Int. J. Greenh. Gas Control.

[12] Amiri M, Shahhosseini S (2018). Optimization of CO_2_ capture from simulated flue gas using K_2_CO_3_/Al_2_O_3_ in a micro fluidized bed reactor. Energy Fuels.

[13] Goli A (2016). An overview of biological processes and their potential for CO_2_ capture. J. Environ. Manage..

[14] Pashaei H, Ghaemi A, Nasiri M (2017). Experimental investigation of CO_2_ removal using Piperazine solution in a stirrer bubble column. Int. J. Greenh. Gas Control.

[15] Stowe HM, Paek E, Hwang GS (2016). First-principles assessment of CO_2_ capture mechanisms in aqueous piperazine solution. Phys. Chem. Chem. Phys..

[16] Pashaei H (2018). Experimental investigation of the effect of nano heavy metal oxide particles in Piperazine solution on CO_2_ absorption using a stirrer bubble column. Energy Fuels.

[17] Hiwale R, Smith R, Hwang S (2015). A novel methodology for the modeling of CO_2_ absorption in monoethanolamine (MEA) using discrimination of rival kinetics. J. Ind. Eng. Chem..

[18] Kim YE (2014). Carbon dioxide absorption using a phase transitional alkanolamine–alcohol mixture. J. Ind. Eng. Chem..

[19] Zhao X (2018). Recent progress of amine modified sorbents for capturing CO2 from flue gas. Chin. J. Chem. Eng..

[20] Fashi F, Ghaemi A, Moradi P (2019). Piperazine-modified activated alumina as a novel promising candidate for CO_2_ capture: Experimental and modeling. Greenh. Gases Sci. Technol..

[21] Moioli S, Pellegrini LA (2016). Modeling the methyldiethanolamine-piperazine scrubbing system for CO_2_ removal: Thermodynamic analysis. Front. Chem. Sci. Eng..

[22] Xu G-W (1998). Gas− liquid equilibrium in a CO_2_− MDEA− H_2_O system and the effect of piperazine on it. Ind. Eng. Chem. Res..

[23] Liu H-B, Zhang C-F, Xu G-W (1999). A study on equilibrium solubility for carbon dioxide in methyldiethanolamine− piperazine− water solution. Ind. Eng. Chem. Res..

[24] Bishnoi S, Rochelle GT (2002). Thermodynamics of piperazine/methyldiethanolamine/water/carbon dioxide. Ind. Eng. Chem. Res..

[25] Böttger A, Ermatchkov V, Maurer G (2009). Solubility of carbon dioxide in aqueous solutions of N-methyldiethanolamine and piperazine in the high gas loading region. J. Chem. Eng. Data.

[26] Speyer D, Ermatchkov V, Maurer G (2010). Solubility of carbon dioxide in aqueous solutions of N-methyldiethanolamine and piperazine in the low gas loading region. J. Chem. Eng. Data.

[27] Najibi H, Maleki N (2013). Equilibrium solubility of carbon dioxide in N-methyldiethanolamine+ piperazine aqueous solution: Experimental measurement and prediction. Fluid Phase Equilib..

[28] Halim H, Shariff A, Bustam M (2015). High pressure CO_2_ absorption from natural gas using piperazine promoted 2-amino-2-methyl-1-propanol in a packed absorption column. Sep. Purif. Technol..

[29] Ume CS, Alper E, Gordesli FP (2013). Kinetics of carbon dioxide reaction with aqueous mixture of piperazine and 2-amino-2-ethyl-1, 3-propanediol. Int. J. Chem. Kinet..

[30] Merajin MT, Sharifnia S, Mansouri A (2014). Process modeling and optimization of simultaneous direct conversion of CO_2_ and CH_4_ greenhouse gas mixture over TiO_2_/webnet photocatalyst. J. Taiwan Inst. Chem. Eng..

[31] Bezerra MA (2008). Response surface methodology (RSM) as a tool for optimization in analytical chemistry. Talanta.

[32] Baş D, Boyacı IH (2007). Modeling and optimization I: Usability of response surface methodology. J. Food Eng..

[33] Ansari F (2016). Application of ZnO nanorods loaded on activated carbon for ultrasonic assisted dyes removal: Experimental design and derivative spectrophotometry method. Ultrason. Sonochem..

[34] Amdoun R (2010). Optimization of the culture medium composition to improve the production of hyoscyamine in elicited *Datura stramonium* L. hairy roots using the response surface methodology (RSM). Int. J. Mol. Sci..

[35] Oliveira R (2006). Experimental design of 2, 4-dichlorophenol oxidation by Fenton's reaction. Ind. Eng. Chem. Res..

[36] Nuchitprasittichai A, Cremaschi S (2013). Optimization of CO_2_ capture process with aqueous amines: A comparison of two simulation-optimization approaches. Ind. Eng. Chem. Res..

[37] Desai KM (2005). Use of an artificial neural network in modeling yeast biomass and yield of β-glucan. Process. Biochem..

[38] Mjalli FS, Al-Asheh S, Alfadala H (2007). Use of artificial neural network black-box modeling for the prediction of wastewater treatment plants performance. J. Environ. Manage..

[39] Zhou Q (2011). Modeling of the carbon dioxide capture process system using machine intelligence approaches. Eng. Appl. Artif. Intell..

[40] Machesa MGK, Tartibu LK, Okwu MO (2023). Performance analysis of stirling engine using computational intelligence techniques (ANN & Fuzzy Mamdani Model) and hybrid algorithms (ANN-PSO & ANFIS). Neural Comput. Appl..

[41] Machesa, M., *et al.* Performance prediction of a stirling heat engine using artificial neural network model. In *2020 International Conference on Artificial Intelligence, Big Data, Computing and Data Communication Systems (icABCD)*. 2020. IEEE.

[42] Mashhadimoslem H (2021). Development of predictive models for activated carbon synthesis from different biomass for CO_2_ adsorption using artificial neural networks. Ind. Eng. Chem. Res..

[43] Piuleac C-G (2012). Hybrid model of a wastewater-treatment electrolytic process. Int. J. Electrochem. Sci.

[44] Curteanu S (2011). Modeling of electrolysis process in wastewater treatment using different types of neural networks. Chem. Eng. J..

[45] Box GE, Wilson KB (1951). On the experimental attainment of optimum conditions. J. Roy. Stat. Soc.: Ser. B (Methodol.).

[46] Liyana-Pathirana C, Shahidi F (2005). Optimization of extraction of phenolic compounds from wheat using response surface methodology. Food chem..

[47] Khodaei B, Sobati MA, Shahhosseini S (2016). Optimization of ultrasound-assisted oxidative desulfurization of high sulfur kerosene using response surface methodology (RSM). Clean Technol. Environ. Policy.

[48] Khodaei B, Sobati MA, Shahhosseini S (2017). Rapid oxidation of dibenzothiophene in model fuel under ultrasound irradiation. Monatshefte für Chemie-Chem. Mon..

[49] Gil M (2013). Response surface methodology as an efficient tool for optimizing carbon adsorbents for CO2 capture. Fuel Process. Technol..

[50] Myers RH, Montgomery DC, Anderson-Cook CM (2016). Response Surface Methodology: Process and Product Optimization Using Designed Experiments.

[51] Zhang H (2017). Highly efficient synthesis of biodiesel catalyzed by CF 3 SO 3 H-functionalized ionic liquids: Experimental design and study with response surface methodology. React. Kinet. Mech. Catal..

[52] Baziar A, Ghashang M (2016). Preparation of pyrano [3, 2-c] chromene-3-carbonitriles using ZnO nano-particles: A comparison between the Box-Behnken experimental design and traditional optimization methods. React. Kinet. Mech. Catal..

[53] Gidiagba, J. O., Tartibu, L., & Okwu. M. O. Crack detection on a structural beam: A simplified analytical method based on artificial neural network model. In *2022 International Conference on Artificial Intelligence, Big Data, Computing and Data Communication Systems (icABCD)*. 2022. IEEE.

[54] Wen Z, Liao W, Chen S (2005). Production of cellulase by Trichoderma reesei from dairy manure. Biores. Technol..

[55] Gidiagba, J. O., Tartibu, L., & Okwu. M.O. Application of soft computing technique based on ANN model prediction in diverse area of mining blasting operations. In *2022 International Conference on Artificial Intelligence, Big Data, Computing and Data Communication Systems (icABCD)*. 2022. IEEE.

[56] Ferreira SC (2007). Box-Behnken design: An alternative for the optimization of analytical methods. Anal. Chim. Acta.

[57] Mayerhoff ZD, Roberto IC, Franco TT (2004). Purification of xylose reductase from Candida mogii in aqueous two-phase systems. Biochem. Eng. J..

[58] Dao DS, Yamada H, Yogo K (2015). Response surface optimization of impregnation of blended amines into mesoporous silica for high-performance CO_2_ Capture. Energy Fuels.

[59] Gil MV, Martínez M, Garcia S, Rubiera F, Pis JJ, Pevida C (2013). Response surface methodology as an efficient tool for optimizing carbon adsorbents for CO_2_ capture. Fuel Process. Technol..

[60] Shafeeyan MS (2012). The application of response surface methodology to optimize the amination of activated carbon for the preparation of carbon dioxide adsorbents. Fuel.

[61] Nuchitprasittichai A, Cremaschi S (2011). Optimization of CO_2_ capture process with aqueous amines using response surface methodology. Comput. Chem. Eng..

[62] Nuchitprasittichai A, Cremaschi S (2013). An algorithm to determine sample sizes for optimization with artificial neural networks. AIChE J..

[63] Morero B, Groppelli ES, Campanella EA (2017). Evaluation of biogas upgrading technologies using a response surface methodology for process simulation. J. Clean. Prod..

[64] Babamohammadi S (2018). Solubility of CO_2_ in aqueous solutions of glycerol and monoethanolamine. J. Mol. Liq..

[65] Sipöcz N, Tobiesen FA, Assadi M (2011). The use of artificial neural network models for CO_2_ capture plants. Appl. Energy.

[66] Basheer IA, Hajmeer M (2000). Artificial neural networks: Fundamentals, computing, design, and application. J. Microbiol. Methods.

[67] Wu Y, Zhou Q, Chan CW (2010). A comparison of two data analysis techniques and their applications for modeling the carbon dioxide capture process. Eng. Appl. Artif. Intell..

[68] Wu Y, Chan CW (2011). Analysis of data for the carbon dioxide capture domain. Eng. Appl. Artif. Intell..

[69] Omoregbee, H., A review of artificial neural network applications in petroleum exploration, production and distribution operations. (2022).

[70] Ewim DRE (2022). A quick review of the applications of artificial neural networks (ANN) in the modelling of thermal systems. Eng. Appl. Sci. Res..

[71] Tan, L.S., *et al.* Application of response surface methodology to investigate CO_2_ absorption column temperature rise. In *Advanced Materials Research*. Trans Tech Publ. (2014).

[72] El-Naas MH (2016). Statistical analysis and optimization of a process for CO_2_ capture. World Acad. Sci Eng. Technol. Int. J. Chem. Mol. Eng.

[73] Mohammad AF (2016). Optimization of a solvay-based approach for CO_2_ capture. Int. J. Chem. Eng. Appl..

[74] Nguyen D-MK (2018). Response surface method for modeling the removal of carbon dioxide from a simulated gas using water absorption enhanced with a liquid-film-forming device. J. Environ. Sci..

[75] Karimi M (2018). CO2 capture in chemically and thermally modified activated carbons using breakthrough measurements: Experimental and modeling study. Ind. Eng. Chem. Res..

[76] Hemmati A (2019). Using rate based simulation, sensitivity analysis and response surface methodology for optimization of an industrial CO_2_ capture plant. J. Nat. Gas Sci. Eng..

[77] Hosseini-Ardali SM (2020). Multi-objective optimization of post combustion CO_2_ capture using methyldiethanolamine (MDEA) and piperazine (PZ) bi-solvent. Energy.

[78] Maleki N, Motahari K (2018). Absorption performance of carbon dioxide in 4-Hydroxy-1-methylpiperidine+ aminoethylethanolamine aqueous solutions: Experimental measurement and modeling. J. Nat. Gas Sci. Eng..

[79] García S (2013). Cyclic operation of a fixed-bed pressure and temperature swing process for CO_2_ capture: Experimental and statistical analysis. Int. J. Greenh. Gas Control.

[80] Ölmez T (2009). The optimization of Cr (VI) reduction and removal by electrocoagulation using response surface methodology. J. Hazard. Mater..

[81] Körbahti BK, Rauf M (2008). Application of response surface analysis to the photolytic degradation of Basic Red 2 dye. Chem. Eng. J..

[82] Khuri AI, Mukhopadhyay S (2010). Response surface methodology. Wiley Interdiscip. Rev. Computat. Stat..

[83] Cullinane, J.T., Thermodynamics and kinetics of aqueous piperazine with potassium carbonate for carbon dioxide absorption. (2005).

[84] Gilmour SG (2006). Response surface designs for experiments in bioprocessing. Biometrics.

[85] Bruns RE, Scarminio IS, de Barros Neto B (2006). Statistical Design-Chemometrics.

[86] Teófilo, R. F., & Ferreira, M. *Quimiometria II: planilhas eletrônicas para cálculos de planejamentos experimentais, um tutorial.* Quim. Nova, (2006).

[87] Montgomery DC, Runger GC (2010). Applied Statistics and Probability for Engineers.

[88] Sadeghi N, Sharifnia S, Trong-On D (2018). Optimization and modeling of CO_2_ photoconversion using a response surface methodology with porphyrin-based metal organic framework. React. Kinet. Mech. Catal..

[89] Mourabet M (2017). Use of response surface methodology for optimization of fluoride adsorption in an aqueous solution by Brushite. Arabian J. Chem..

[90] Amiri M, Shahhosseini S, Ghaemi A (2017). Optimization of CO_2_ capture process from simulated flue gas by dry regenerable alkali metal carbonate based adsorbent using response surface methodology. Energy Fuels.

[91] Wang C (2016). Application of response surface methodology to the chemical cleaning process of ultrafiltration membrane. Chin. J. Chem. Eng..

[92] Zhang Y-J (2012). Optimization of succinic acid fermentation with Actinobacillus succinogenes by response surface methodology (RSM). J. Zhejiang Univ. Sci. B.

[93] Dixon B, Candade N (2008). Multispectral landuse classification using neural networks and support vector machines: One or the other, or both?. Int. J. Remote Sens..

[94] Hebb DO (1949). The Organization of Behaviour: A Neuropsychological Theory.

[95] Grossberg ST (2012). Studies of Mind and Brain: Neural Principles of Learning, Perception, Development, Cognition, and Motor Control.

[96] Hopfield JJ (1982). Neural networks and physical systems with emergent collective computational abilities. Proc. Natl. Acad. Sci..

[97] Widrow, B., Winter, R., & Baxter, R. A. Learning phenomena in layered neural networks. In *Proceedings of the IEEE First International Conference on Neural Networks*. (1987).

[98] Siddique N, Adeli H (2013). Computational Intelligence: Synergies of Fuzzy Logic, Neural Networks and Evolutionary Computing.

[99] Richards JA, Richards J (1999). Remote Sensing Digital Image Analysis.

[100] Foresee, F. D., & Hagan, M. T.Gauss-Newton approximation to Bayesian learning. In *Proceedings of International Conference on Neural Networks (ICNN'97)*. (1997). IEEE.

[101] Fausett LV (2006). Fundamentals of Neural Networks: Architectures, Algorithms and Applications.

[102] Kobayashi K, Salam MU (2000). Comparing simulated and measured values using mean squared deviation and its components. Agron. J..

[103] Hagan MT, Menhaj MB (1994). Training feedforward networks with the Marquardt algorithm. IEEE Trans. Neural Networks.

[104] Ticknor JL (2013). A Bayesian regularized artificial neural network for stock market forecasting. Expert Syst. Appl..

[105] Møller MF (1993). A scaled conjugate gradient algorithm for fast supervised learning. Neural Netw..

[106] Piñeiro G (2008). How to evaluate models: Observed vs. predicted or predicted vs. observed?. Ecol. Modell..

