# Supplementary material for: Modeling and optimization of CO2 mass transfer flux into Pz-KOH-CO2 system using RSM and ANN
Source: Sci Rep. 2023 Mar 10;13:4011. doi: 10.1038/s41598-023-30856-w (PMC10006194; doi:10.1038/s41598-023-30856-w)
Supplement: Supplementary file 2 — Supplementary Information 2. [file 41598_2023_30856_MOESM2_ESM.docx]

**Supplementary**


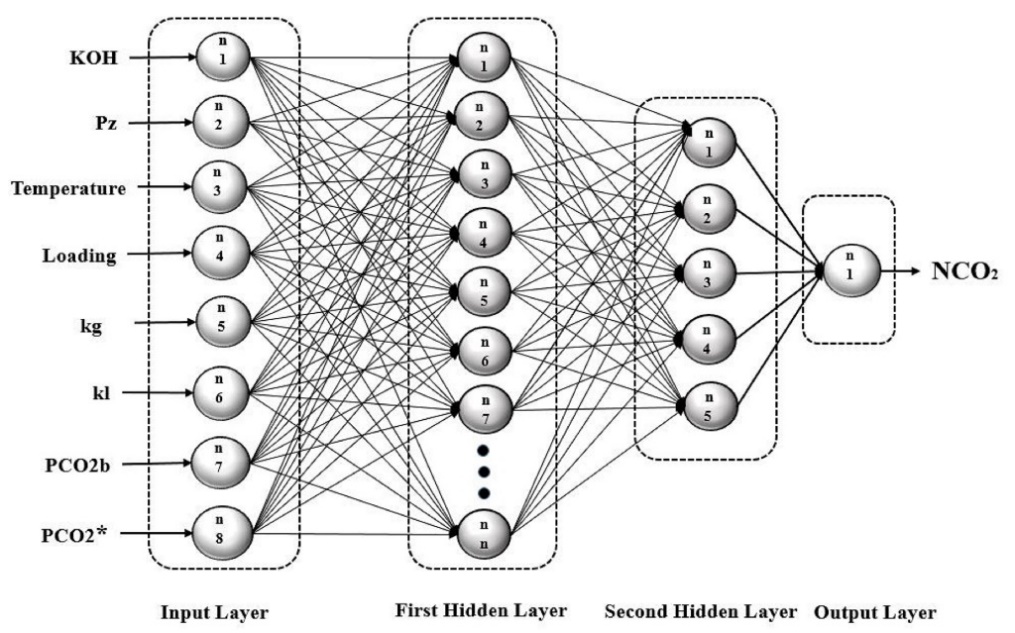


**Figure S1.** The absorption process structure *ANN* of feed forward *MLP* with backpropagation**.**

**
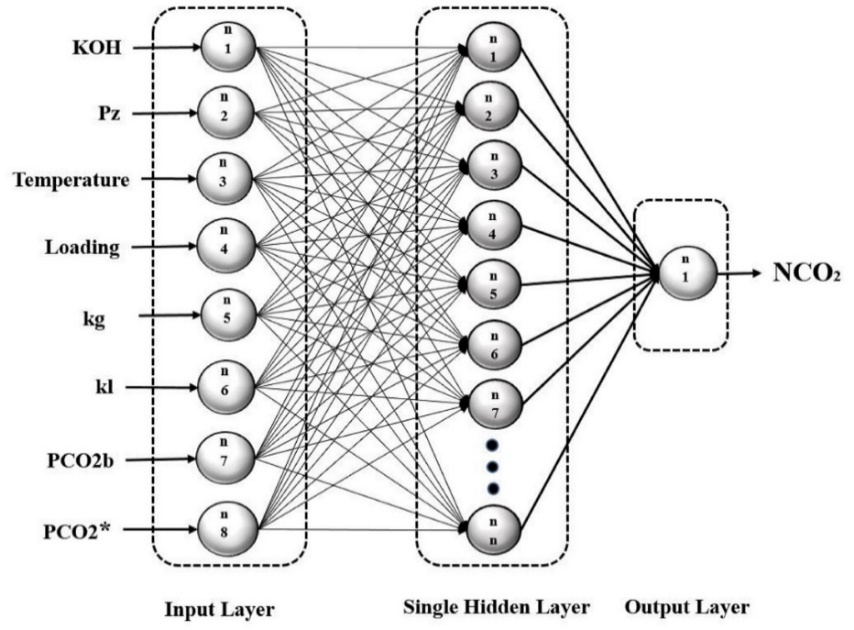
**

**Figure S2.** The absorption process *ANN* structure of *RBF*.

**
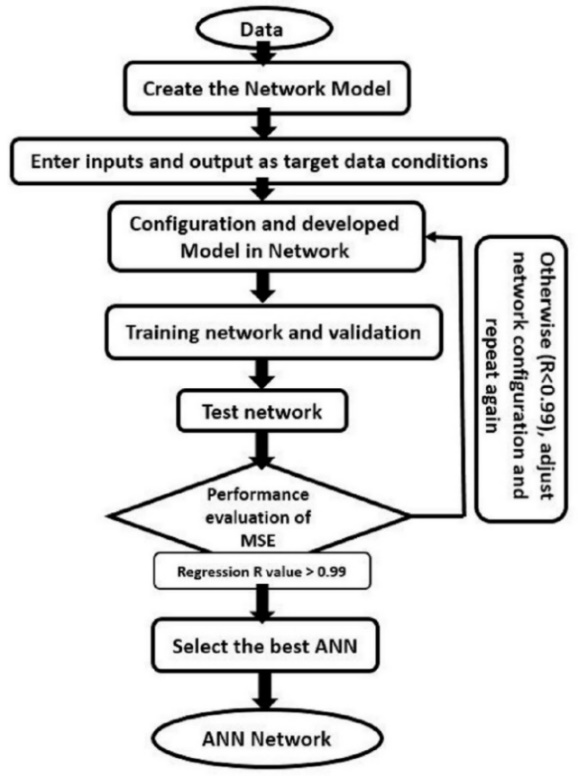
**

**Figure S3.** Schematic flowchart diagram of the $ANN$ structure design

| **(a)** | **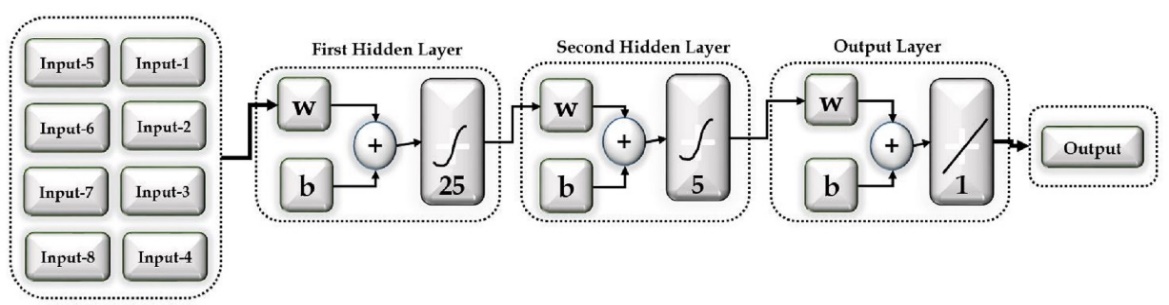** |
| --- | --- |
| **(b)** | **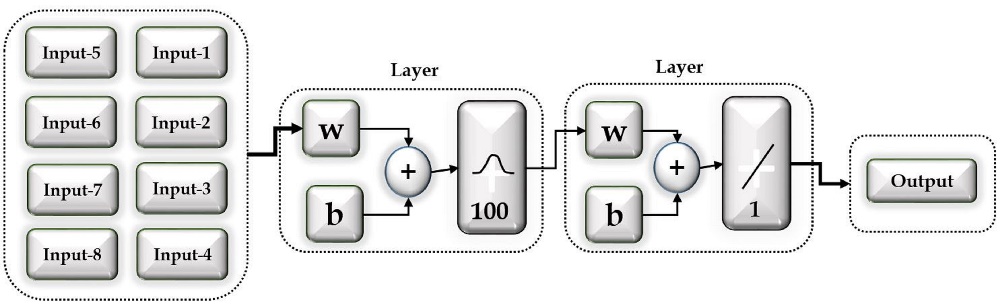** |

**Figure S4.** *ANN* structure of (a) *MLP* and (b) *RBF* models for absorption process

**Table S1.** Level and range of independent variables for *CCD* runs

| **Factors** | **Name** | **Symbol** | **Units** | **Level** | | | | |
| --- | --- | --- | --- | --- | --- | --- | --- | --- |
|  |  |  |  | **-α^1^** | **-1** | **0** | **+1** | **+α^1^** |
| *KOH* concentration | *KOH* | X_1_ | mol/l | 2.5 | 3.42 | 4.37 | 5.30 | 6.22 |
| *Pz* concentration | *Pz* | X_2_ | mol/l | 0.6 | 1.35 | 2.1 | 2.85 | 3.6 |
| Temperature | T | X_3_ | C | 10.1 | 35.5 | 60.9 | 86.3 | 111.7 |
| Loading | loading | X_4_ | mol/mol | 0.262 | 0.387 | 0.511 | 0.636 | 0.761 |
| Gas phase mass transfer coefficient | $k_{g}\times10^{9}$ | X_5_ | kmol/pa.m^2^.s | 1.63 | 9.55 | 17.46 | 25.38 | 33.3 |
| Liquid phase mass transfer coefficient | $k_{l}\times10^{4}$ | X_6_ | m/s | 0.58 | 0.795 | 1.01 | 1.225 | 1.44 |
| Gas bulk partial pressure | $p_{CO_{2},b}$ | X_7_ | Pa | 0 | 15014 | 30025 | 45042 | 60056 |
| equilibrium partial pressure | $p_{CO_{2}}^{*}$ | X_8_ | Pa | 0 | 9178.5 | 18357 | 27535.5 | 36714 |
| Response | $N_{CO_{2}}\times10^{7}$ | X_9_ | kmol/m^2^ s | -107.7 | -2.55 | 102.6 | 207.72 | 312.85 |

α= 2 (pivot point for orthogonal *CCD* about eight independent variables).

**Table S2.** Communication between actual and coded values of the variables

| **Code** | **Actual level of variable** |
| --- | --- |
|  |  |
| -α | **$\xi_{\min}$** |
| -1 | **$[(\xi_{\min}+\xi_{\max})/2]+[(\xi_{\min}-\xi_{\max})/2\beta]$** |
| 0 | **$(\xi_{\min}+\xi_{\max})/2$** |
| +1 | **$[(\xi_{\min}+\xi_{\max})/2]-[(\xi_{\min}-\xi_{\max})/2\beta]$** |
| +α | **$\xi_{\max}$** |
| ****and **$\xi_{\max}$** are the minimum and maximum values of **$\xi$** respectively; β is 2^n/4^ | |

The numbers of the degree of freedom for these sources are represented in Supplementary Table [S1](https://www.nature.com/articles/s41598-023-29250-3#MOESM3), where *p* is the number of coefficients of the mathematical model, *m* represents the number of levels used and *n* represents the number of total observations.

**Table S3.** *AVOVA* for a mathematical model fitted to an experimental data

| **Variation source** | **Sum of the square** | **Degree of freedom** |
| --- | --- | --- |
| Regression | $SS_{reg}=\sum_{i}^{m} \sum_{j}^{n1} (\hat{y}_{i}-\bar{y})^{2}$ | p-1 |
| Residuals | $SS_{res}=\sum_{i}^{m} \sum_{j}^{n1} (y_{ij}-\hat{y}_{i})^{2}$ | n-p |
| Lack of fit | $SS_{lof}=\sum_{i}^{m} \sum_{j}^{n1} (\hat{y}_{i}-\bar{y}_{i})^{2}$ | m-p |
| Pure error | $SS_{pe}=\sum_{i}^{m} \sum_{j}^{n_{i}} (y_{ij}-\bar{y}_{i})^{2}$ | n-m |
| Total | $SS_{tot}=\sum_{i}^{m} \sum_{j}^{n_{i}} (y_{ij}-\bar{y})^{2}$ | n-1 |
| *m*, total levels number in the plan; *n_i_*, observations number; *p*, number of model parameter; ȳ, overall media; $\hat{y}_{i}$, estimated value for the level *i* by the model; ȳ_i_ , repeats media carried out in the same set of empirical conditions. *y_ij_*, repeats performed in each single levels | | |
